# M6 Membrane Protein Plays an Essential Role in *Drosophila* Oogenesis

**DOI:** 10.1371/journal.pone.0019715

**Published:** 2011-05-16

**Authors:** María Paula Zappia, Marcela Adriana Brocco, Silvia C. Billi, Alberto C. Frasch, María Fernanda Ceriani

**Affiliations:** 1 Instituto de Investigaciones Biotecnológicas, Instituto Tecnológico de Chascomús, Consejo Nacional de Investigaciones Científicas y Técnicas, Universidad Nacional de General San Martín, Buenos Aires, Argentina; 2 Laboratorio de Genética del Comportamiento, Fundación Instituto Leloir and Instituto de Investigaciones Bioquímicas - Buenos Aires (IIB-BA, CONICET), CABA, Buenos Aires, Argentina; University of Texas MD Anderson Cancer Center, United States of America

## Abstract

We had previously shown that the transmembrane glycoprotein M6a, a member of the proteolipid protein (PLP) family, regulates neurite/filopodium outgrowth, hence, M6a might be involved in neuronal remodeling and differentiation. In this work we focused on M6, the only PLP family member present in *Drosophila*, and ortholog to M6a. Unexpectedly, we found that decreased expression of M6 leads to female sterility. M6 is expressed in the membrane of the follicular epithelium in ovarioles throughout oogenesis. Phenotypes triggered by *M6* downregulation in hypomorphic mutants included egg collapse and egg permeability, thus suggesting M6 involvement in eggshell biosynthesis. In addition, RNAi-mediated *M6* knockdown targeted specifically to follicle cells induced an arrest of egg chamber development, revealing that M6 is essential in oogenesis. Interestingly, M6-associated phenotypes evidenced abnormal changes of the follicle cell shape and disrupted follicular epithelium in mid- and late-stage egg chambers. Therefore, we propose that M6 plays a role in follicular epithelium maintenance involving membrane cell remodeling during oogenesis in *Drosophila*.

## Introduction

The glycoprotein M6a was identified years ago, and despite its prominent expression in the central nervous system and other tissues [1], [2], its biological function is still unknown. Previous findings established that chronic stress decreases *M6a* mRNA levels in the hippocampus, and this downregulation is prevented by chronic administration of antidepressants [3], [4]. In addition M6a plays an important role in neurite outgrowth and filopodium/spine formation [5], as well as in filopodium motility and likely synapse formation [6], suggesting that it might be involved in the plastic changes found in the hippocampus of stressed/antidepressant-treated animals. M6a was also found to be involved in the differentiation of neurons derived from embryonic stem cells [7]. In addition to its neuronal expression, M6a is also expressed in different epithelial cell types, such as the proximal tubules of the kidney, the choroid plexus [1], [8], and human lung and ovary (www.genecards.org).

M6a belongs to the myelin proteolipid protein (PLP) family. In mammals, other members of this family include the closely related M6b and the founder PLP, with its splice variant DM20. All of the PLP members have four transmembrane domains that allow their localization at the plasma membrane and are widely conserved along evolution from arthropods to mammals [9], [10]. Interestingly, other members of the PLP family such as M6b and DM20, but not PLP, are also regulated by chronic stress, and were shown to be involved in neurite outgrowth and filopodium formation [11]. Members of the PLP family were proposed to interact with the actin cystoskeleton after their association with actin-enriched membranes [12]. Additionally, PLP and DM20 were proposed to act as adhesion molecules [13], and M6a as an ion channel [14], underscoring that the molecular function of the PLP family remains undefined.

While mammals contain three *plp* genes (*M6a*, *M6b* and *Plp1*), a single *M6* gene is found in arthropods [10]. Not only the structural organization of the fly *M6* gene resembles that of the mouse *M6a* gene, but also the predicted M6 protein shares amino acid similarity with the mouse M6a [15], [16], suggesting that M6 might be the functional M6a homolog. However, up to date no experimental evidence for M6 expression or function has been reported. Thus we set forward to investigate M6a requirement employing *Drosophila* as the model system.

In this work we focused on a phenotype, namely female sterility in *D. melanogaster*, induced by *M6* downregulation using hypomorphic alleles and RNAi lines. Such sterility was associated with egg permeability and collapse, suggesting M6 involvement in eggshell biosynthesis. We found that M6 is expressed in the follicular epithelium of egg chambers throughout oogenesis in wild type flies. Tissue-specific M6 knockdown targeted to follicle cells impaired follicle cell shape changes and triggered disruption of the follicular monolayer. Indeed, partial loss of M6 function unraveled its key role at stages where cell adhesion, cytoskeletal rearrangements and cell-cell communication enable cell shape changes and organization.

## Materials and Methods

### Fly strains

Flies were grown and maintained at 25°C in vials containing standard cornmeal-agar medium.

A *w^1118^* stock was used as the wild-type control. Potential *M6* (CG 7540) mutant stocks *y^1^ w^67c23^*; P{EPgy2}M6^EY07032^, *w^1118^*; P{GT1}M6^BG00390^ and *w^1118^*; Mi{ET1}M6^MB02608^/TM3, Sb^1^ Ser^1^ were renamed *M6* ^01^, *M6* ^02^ and *M6* ^03^, respectively, and were obtained from the Bloomington Stock Center, along with the GAL80^ts^ lines. The CA06602 stock (*M6* ^GFP^) was obtained from the GFP Protein Trap Database at the Carnegie Institution [17]. The *M6* ^01^, *M6* ^02^ and *M6* ^GFP^ strains were backcrossed several generations to *w^1118^* to minimize background effects. The follicular GAL4 driver PG45.G4/FM7 was provided by Dr. Stein. Fly strains, such as 198Y-GAL4 (P{GawB}198Y), e22c-GAL4 (P{en2.4-GAL4}e22c), 55B-GAL4 (P{GawB}55B), da.G32-GAL4 (P{GAL4-da.G32}UH1), slbo.2.6-GAL4 (P{GAL4-slbo.2.6}1206), c355-GAL4 (P{GawB}c355), c204-GAL4 (P{GawB}c204), T155-GAL4 (P{GawB}T155), c329b-GAL4 (P{GawB}c329b), tubP-GAL4 (P{tubP-GAL4}LL7), and UAS-CD8GFP were obtained from the Bloomington Stock Center. The UAS-*M6*-RNAi strains employed were: ID 7540R-1 and 7540R-2 from the National Institute of Genetics Fly Stock Center (NIG-Fly) termed here as *M6*-RNAi_NIG_ (only the first one was shown), and 101757 from the KK-library Vienna Drosophila RNAi Center (VDRC) termed here as *M6*-RNAi_VDRC_. Experiments with the TARGET system [18] were carried out at 18°C to repress GAL4-mediated transcriptional activation. The system was induced at 29°C or 32°C (as indicated). To switch it off flies were transferred to 18°C. After 7 days a complete rescue of the original phenotype was achieved.

*M6* mutant alleles were generated by excision. The original P-element (EY07032) from *M6* ^01^ was removed with the transposase (Δ2–3). White-eyed stocks corresponding to independent excision events were kept as *M6* ^Δ01^ allele strains. To characterize the *M6* allelic series, PCR reactions on genomic DNA from homozygous flies were performed as detailed in Table S1. PCR and sequencing reactions allowed the identification of imprecise excisions.

### Fertility experiments

Female fertility was assessed crossing two females (or 4 females, if at 18°C) to 5 wild type males per vial. For male fertility experiments, 5 males were crossed to 2 wild type females. Crosses were kept in each vial with yeast paste for 2–3 days at 25°C when testing the fertility of *M6* alleles, or at 18/29°C in *M6* knockdown experiments. The number of adult offspring per vial resulting from each cross was quantified.

### Eggshell analysis

Egg laying was analyzed in batches of 30 or 50–60 females with wild type males when incubated at 25°C or 18/29°C respectively. Females were allowed to lay eggs on yeasted agar plates for 6 hours at 25°C or for 4 hours at 18/29°C. Only eggs laid during the afternoon were scored in order to reduce variability due to rhythmic behavior [19]. The number of eggs laid in each plate was normalized by the number of tested females.

Egg collapse was determined collecting 0–4 hours-old eggs and transferring them onto dry plates. Collapsed and non-collapsed eggs were counted every 5 minutes.

Eggshell permeability was assessed by the neutral red assay (adapted from [20], [21]). Briefly, laid eggs (0–4 hours-old) were collected and incubated in 5 mg/ml neutral red in PBS for 1 h. Eggs were washed three times and mounted for visualization. Only colored eggs indicating neutral red uptake were scored as permeable. Only intact eggs were analyzed.

### Immunofluorescence and image analysis

Ovaries from well-fed females were dissected, fixed and stained as described elsewhere [20]. Briefly, ovarioles were dissected and fixed in 5% formaldehyde in PB for 10 minutes. Tissue was permeabilized with 0.3% Triton X-100 in PB with or without 0.3% sodium deoxycholate for 15 minutes. Blocking solution was 2% bovine serum albumin (FE.DE.SA, Argentina) and 5% goat serum (Natocor). Tissues were blocked for 1 h. Primary antibodies were diluted in blocking solution and incubated overnight at 4°C. Washes were carried out in 0.3% Triton X-100 in PBS for 20 minutes and repeated 3 times. Tissues were blocked for 1 hour before incubation with secondary antibodies. Ovaries were incubated with secondary antibodies for 2 h. All steps were carried out at room temperature otherwise indicated. Ovaries were stored in FluorSave Reagent (Calbiochem) overnight at 4°C and then mounted. Primary antibodies employed throughout the work for ovary immunostaining were mouse α-Armadillo (N2-71A1, 1∶100; Developmental Studies Hybridoma Bank (DSHB)), mouse α-Fasciclin III (7G10,1∶100 DSHB), polyclonal guinea pig α-Cad99C (GP5, 1∶3000 [20], provided by Dr. D. Godt), rabbit α-GFP (1∶300 or 1∶500, Molecular Probes, Invitrogen), rat α-Yolkless (#156, 1∶750, [22]; provided by Dr. A. Cyrklaff). Dr. G. Waring provided rabbit polyclonal sV23 and sV17 antibodies [23]. The sV23 and sV17 antibodies were previously preabsorbed against fixed, devitellinized wild type embryos and used in a 1∶800 dilution. Fixed ovary cryosections were used as described previously [24], [25] for immunostaining of the eggshell proteins sV17 and sV23 because of poor access to the vitelline membrane in whole-mount preparations. Secondary antibodies conjugated to Cy2 or Cy5 were used (1∶500, Jackson ImmunoReasearch Laboratories). Rhodamine-phalloidin and DAPI (1∶300 and 1∶3000, respectively; Molecular Probes, Invitrogen) were used. Fluorescent images were acquired with the laser scanning confocal microscopes (Zeiss LSM510 Meta and Zeiss Pascal) using 20x/0.8, 40x/1.3, 63x/1.4, 100x/1.4 objectives. For egg chamber staging, the length of mid sagittal sections was measured according to Spradling (1993). Images were processed using Photoshop and Illustrator (Adobe Systems).

### mRNA Isolation and Quantitative Real Time Reverse Transcription Polymerase Chain Reaction (RT-qPCR)

Ovaries were dissected from well-fed young females and homogenized in Trizol Reagent (Life Technologies, New York, USA) to isolate total RNA. Then PolyA+mRNA was purified using the PolyATract mRNA Isolation System (Promega, Madison, Wisconsin, USA). Complementary DNA was synthesized using oligo dT and SuperScript™ II Reverse Transcriptase enzyme (Life Technologies).

qPCRs were carried out in a 7500 Real-Time PCR System (Applied Biosystems, Foster City, California, USA). Quantitation of each cDNA was achieved using SYBR Green PCR Master Mix (Applied Biosystems) in triplicate. Primer sequences are detailed in Table S1. Normalization was accomplished employing *Rp49* and *gapdh* as housekeeping genes. Values shown in all figures were calculated using *Rp49* as the reference gene. Normalizations to *gapdh* resulted in almost identical patterns. Relative quantification was performed using a comparative CT method [26], [27]. Before each experiment, the calibration curves were validated. Samples whose curves amplified out of the calibrated dynamic range were eliminated. All procedures followed the manufacturer's instructions. Values shown are relative to the control genotype (white bar) at each temperature condition.

### Statistical data analysis

Graphs were generated with GraphPad Prism version 5.0 (Graphpad Software, California, USA). Statistical analysis was performed with IS (Infostat software). Group means were analyzed for overall statistical significance by unpaired Student's *t* test or one-way analysis of variance (ANOVA) followed by Bonferroni's multiple comparison tests. Non-parametric analysis was performed (Mann-Whitney or Kruskal-Wallis followed by Dunn's Multiple Comparison Test) when assumptions on the normal distribution and variance did not allow otherwise.

## Results

### Reduced *M6* mRNA levels results in female sterility

**Figure 1:**
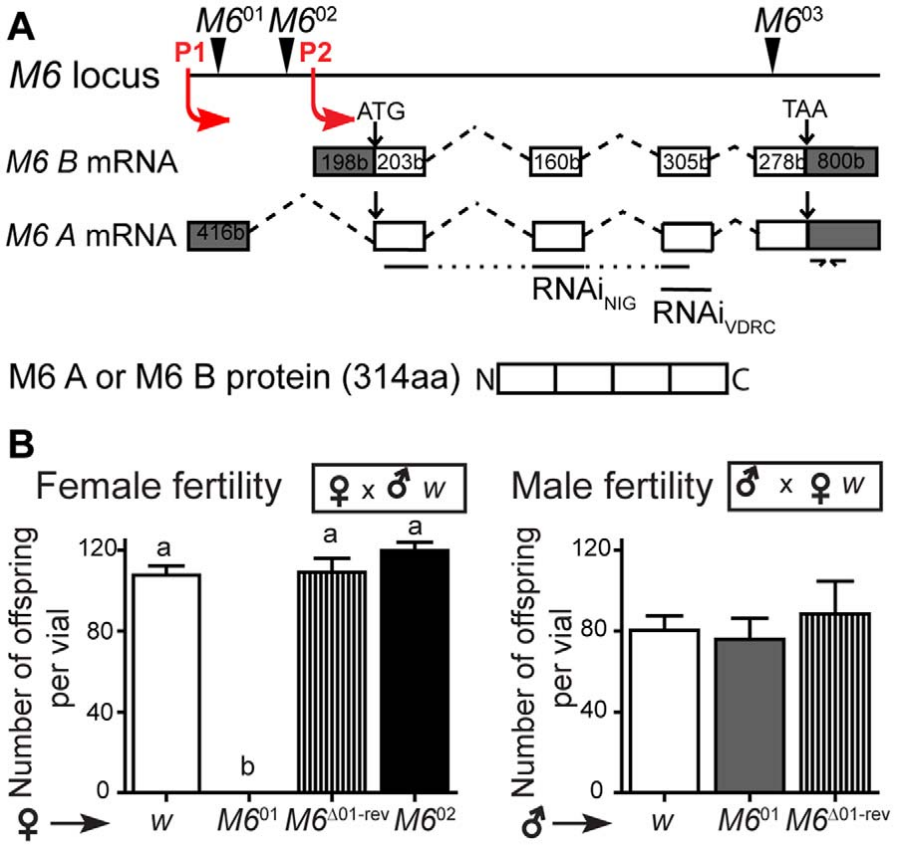
*M6* ^01^ displays female fertility defects. (A) Schematic diagram of the *M6* locus showing the mRNA isoforms (A and B) transcribed from promoters P1 and P2. Exons and introns are indicated by boxes and dashed lines, respectively. Grey boxes represent untranslated regions (UTR) and white boxes highlight coding regions. Both isoforms are translated into the same protein (M6 A/B-314 residues). The location of independent P-element insertions resulting in potential *M6* mutants is indicated by arrowheads (*M6* ^01^ for M6^EY07032^, *M6* ^02^ for M6^BG00390^ and *M6* ^03^ for M6^MB02608^). Lines point to sequences targeted by UAS-*M6-*RNAi stocks. The representation is not scaled. Primers employed to quantify *M6* mRNA levels by RT-qPCR are indicated by arrows in the 3′UTR. (B) Fertility assessment of potential *M6* mutants. Female and male fertility was independently measured as the number of offspring per vial obtained when crossed to wild type flies (*w*), as indicated in the figure. The sterility phenotype of *M6* ^01^ females was reverted by P-element excision. The excised line, termed *M6* ^Δ01-rev^, still contained 50 bp from the original P-element. Mean ± s.e.m., n = 2–3; Kruskal-Wallis test, *p*<0.001 for female fertility, *p*>0.05 for male fertility. Different letters indicate significant differences (*p*<0.01).

To gain insight into M6 function transposon insertions within the *M6* locus were identified (at http://flybase.org/); three potential *M6* mutants, renamed *M6* ^01^, *M6* ^02^ and *M6* ^03^ (Fig. 1A), were extensively analyzed. We focused on *M6* ^01^, which displayed clear fertility defects as homozygotes, while *M6* ^02^ were fertile and *M6* ^03^ were lethal. The lethality phenotype was also observed with other *M6* alleles (data not shown) and ubiquitous expression of *M6*-RNAi (Fig. S6 and Table S2), thus revealing an essential contribution of M6 during *Drosophila* development. To study the sterility phenotype, the number of offspring emerged from crosses involving homozygous *M6* ^01^ or *M6* ^02^ with wild type flies were quantified (Fig. 1B). *M6* ^01^ females were completely sterile while *M6* ^01^ males showed normal fertility. We also observed female sterility of the trans-heterozygote *M6* ^01^/*M6* ^03^ (data not shown). To confirm that female sterility was associated to the insertion within the *M6* locus we mobilized the P-element from *M6* ^01^, giving rise to *M6* ^Δ01-rev^. Excision of *M6* ^01^ rescued fertility to wild type levels (Fig. 1B, left panel), suggesting that altered M6 levels could be responsible for the sterility phenotype.

**Figure 2:**
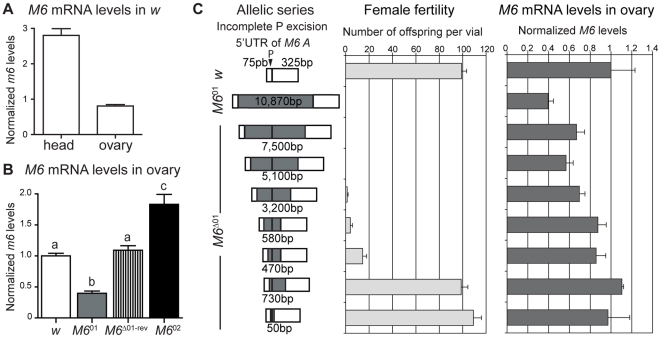
A subtle reduction in *M6* mRNA levels is enough to trigger female sterility. (A) *M6* mRNA levels were measured in ovaries and heads from control flies by RT-qPCR. Mean ± s.e.m., n = 3; unpaired *t*-test with Welch correction, *p*<0.01. (B) *M6* mRNA levels in ovaries of control, *M6* ^01^ and *M6* ^02^ mutants assessed by RT-qPCR. Excision of the original *M6* ^01^ insertion restored *M6* mRNA levels (*M6* ^Δ01-rev^). Mean ± s.e.m., n = 3; logarithmic transformation of the data followed by ANOVA, *p*<0.001. Different letters indicate significant differences (*p*<0.001). (C) Correlation between the degree of female sterility and the remaining *M6* mRNA levels in ovaries of the allelic series. Left panel: Imprecise P-element excision generated an allelic series (*M6* ^Δ01^) spanning seven novel *M6* alleles. White boxes represent the 5′ UTR of *M6-A*; the original P-element insertion in *M6* ^01^ (75 bp of the 5′UTR initiation site; black arrowhead) and the remaining transposon sequences in the excised alleles are shown in grey. Residual P-element were mapped by genomic PCR and DNA sequencing (see Table S1). The size of the remaining fragment is indicated. Middle panel: Female fertility was measured as in Figure 1; mean ± s.e.m., n = 3. Right panel: *M6* mRNA levels in ovaries was measured by RT-qPCR and normalized; mean ± s.e.m., n = 3. Note that two alleles restored *M6* mRNA to wild type levels and fully rescued the female phenotype. Spearman correlation coefficient between female fertility and *M6* mRNA levels is r_s_ = 0.92.

We anticipated that M6 should be present in the central nervous system based on information available on M6a [1], [8]; in addition, the sterility phenotype suggested that M6 is expressed in the ovary. We analyzed *M6* mRNA profiles by RT-qPCR (Fig. 2A) and found that it is expressed in control ovaries but to a lower extent compared to fly heads; moreover, *M6* ^01^ exhibited a significant reduction in *M6* mRNA levels (Fig. 2B), revealing its hypomorphic nature, which were restored upon P-element excision (*M6* ^Δ01-rev^). Interestingly, ovaries from *M6* ^02^, the non-sterile strain, displayed elevated *M6* levels.

The locus contiguous to *M6*, CG33214, is only 384 bp away from the insertion site of the P-element in *M6* ^01^. However, CG33214 mRNA levels in ovaries showed no differences between *M6* ^01^, *M6* ^Δ01-rev^ and control flies by RT-qPCR (Fig. S1), ruling out CG33214 contribution to the *M6* ^01^ phenotype.

Incomplete excision of the P-element in *M6* ^01^ retrieved an allelic series, *M6* ^Δ01^. We analyzed female fertility and *M6* mRNA levels in the ovaries from each *M6* allele. Each novel *M6* allele has a remnant P-element inserted (Fig. 2C, left panel). As *M6* mRNA levels decreased in the allelic series, a drastic decline in the number of progeny was evidenced (middle panel), indicating that subtle changes in *M6* levels (right panel) associated with a severe decrease in female fertility. Male fertility did not show significant differences among *M6* alleles (data not shown). Taken together, these results show that female sterility is triggered by *M6* downregulation.

### Female sterility is accompanied by abnormally permeable eggs

**Figure 3:**
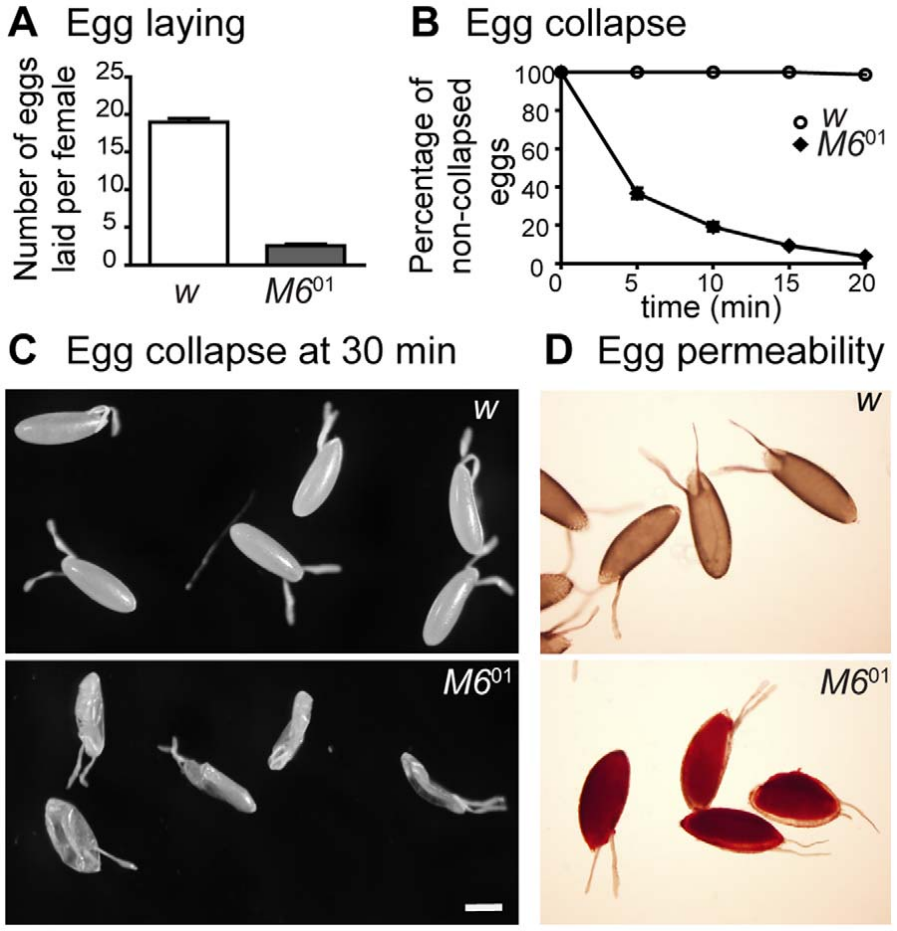
Female sterility results from eggshell malformation. (A) Egg laying was evaluated as the number of eggs laid by control and *M6* ^01^ females crossed to wild type males. Mean ± s.e.m., n = 2; Mann Whitney test, *p*<0.01. (B) Resistance to water loss was determined over time. Eggs laid by control and *M6* ^01^ females were collected, transferred to a dry plate and monitored over time. Collapsed eggs were quantified every five minutes for 20 minutes; the percentage of non-collapsed eggs was plotted, n = 2. (C) Representative images of control and *M6* ^01^ eggs taken 30 minutes into the time course shown in B. Scale bar is 400 µm. (D) Eggs laid by control and *M6* ^01^ females were collected and incubated with the neutral red dye. A total of 100% of *M6* ^01^ mutant eggs were permeable, indication of an abnormal vitelline membrane. Note that *M6* ^01^ eggs become unusually enlarged in aqueous solution.

To explore the basis of the sterility phenotype, we examined the egg laying ability of *M6* ^01^ females. The number of eggs laid by *M6* ^01^ flies was significantly reduced compared to controls (Fig. 3A). Upon close inspection, mutant eggs also exhibited a collapsed structure soon after deposition. We followed in time the percentage of collapsed eggs in *M6* ^01^ and controls (Fig. 3B,C). While control eggs were completely normal, the percentage of collapsed eggs deposited by *M6* ^01^ females increased drastically over time and reached almost 100% in 20 minutes. Using light microscopy, no gross alterations in chorionic structures were observed. However, since collapsed eggs suggest that eggshells -normally restricting permeability and preventing desiccation [20], [21]- are compromised, we assessed egg permeability. Not surprisingly, all mutant eggs became fully stained with the dye neutral red, in contrast to control ones (Fig. 3D). Egg collapse and egg permeability were also evidenced in the trans-heterozygote *M6* ^01^/*M6* ^03^ (Fig. S3A, and data not shown). Permeable eggs are indicative of defects in the integrity of the vitelline membrane, which disrupt the morphogenesis of the wax layer that surrounds it [21]. These results indicate that reduced *M6* mRNA levels induce eggshell malformation, supporting a role for M6 during oogenesis.

**Figure 4:**
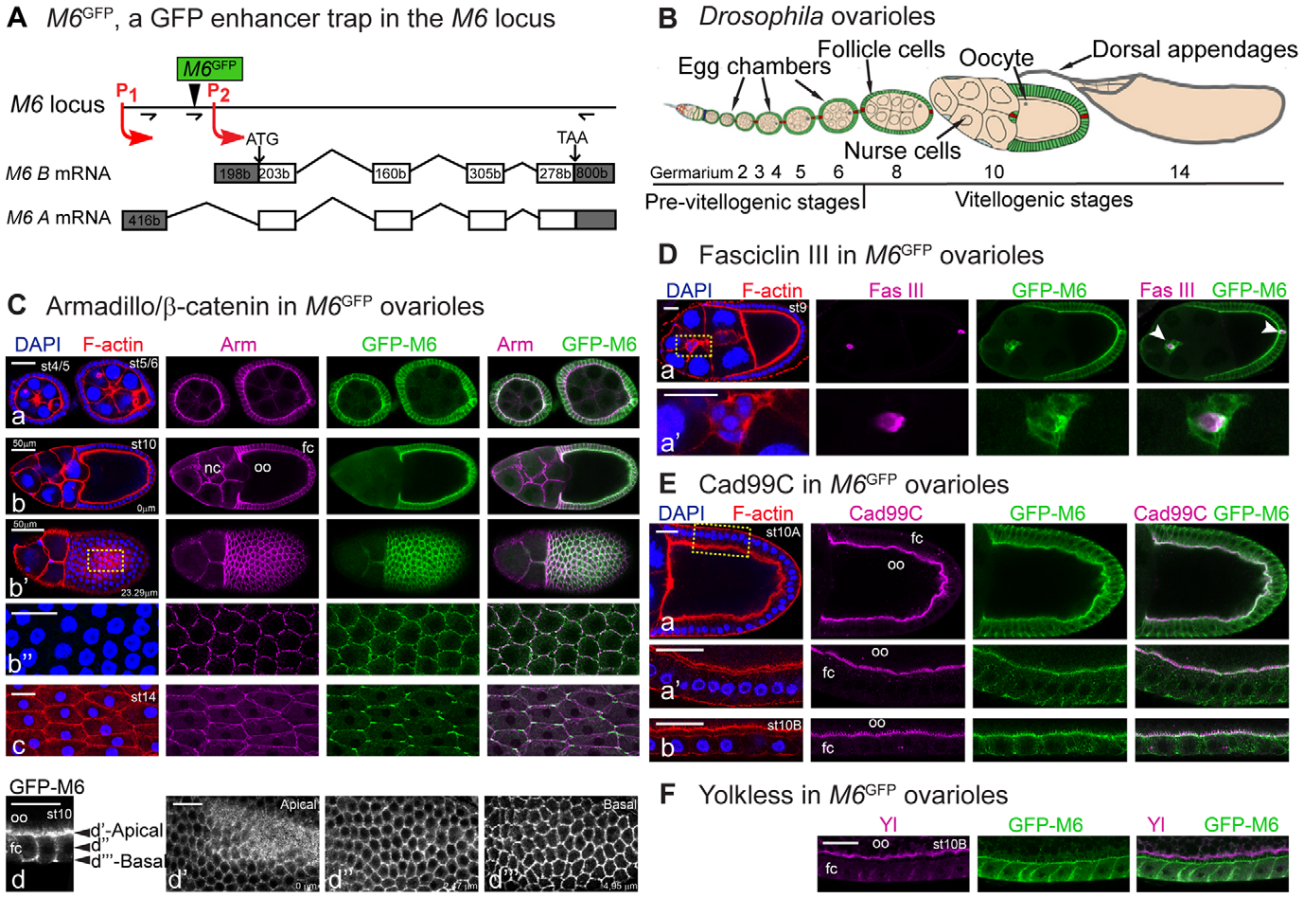
GFP-M6 is restricted to the membrane of the follicular epithelium. (A) M6 localization was investigated using *M6* ^GFP^ (M6^CA06602^). The P-element is indicated (black arrowhead). RT-PCR primers are indicated by arrows (see Table S1). All *M6* isoforms derived from P1 are tagged to GFP at the N-terminus (Zappia *et al.*, unpublished data). (B) Schematic diagram of the *Drosophila* ovariole (adapted from [53]). A monolayer of follicle cells (green) derived from somatic stem cells surround the germ-line cyst (yellow). Polar cells are labeled in red. Developmental stages are indicated (2 to 14). (C–F) Follicular markers were used to analyze GFP-M6 localization. Throughout this work representative images are shown and numbers in each panel indicate developmental stages determined by F-actin (Phallodin, red) and nuclei (DAPI, blue) staining (left panels). The anterior-posterior axis is oriented left to right. (C) Armadillo/β-catenin (magenta) labels the membrane of follicle and nurse cells. GFP-M6 is localized to the membrane of the FE (green). (a–c) Egg chambers from early (a), mid (b-b″) and late (c) oogenesis. Apical/basal (b, 0 µm) and top (b′, 23.29 µm) views of a st10 egg chamber are shown. (b″) A magnified view of the area indicated in b′. Note the change in GFP-M6 distribution within the membrane at later stages (b″ and c). (d-d′″) Apical/basal view of st10 follicle cells (d), and sections corresponding to an apical (d′, 0 µm), middle (d″, 2.47 µm) and basal (d′″, 4.95 µm) membrane domains. (D) GFP-M6 localizes to polar and border cells (white arrowheads), the former labeled with anti-Fasciclin III (magenta); (a′) is a magnified view of the region indicated in a. (E) GFP-M6 localizes to the microvilli extending from the FC apical membrane, visualized with anti-Cad99C antibody (magenta); apical/basal views of the FC from st10A and st10B are shown (a-a′ and b, respectively), along with a magnified view of the indicated region (a′). (F) GFP-M6 is not expressed in the oocyte cortex, stained with anti-Yolkless antibody (magenta). Images (Cb″ and Ea′-b) were processed with the Adaptive PSF Deconvolution method. Scale bar is 20 µm, unless otherwise indicated.

We then focused on the structure of *M6* ^01^ ovarioles. Each ovariole includes a series of egg chambers organized in progressive stages from the germarium to stage 14 that mature into an egg (Fig. 4B). F-actin and DAPI staining did not reveal morphological defects at any stages of oogenesis in *M6* ^01^ (Fig. S2; data not shown). We also employed general markers of oogenesis such as Armadillo/β-catenin to visualize cell-cell adhesive junctions [28], and Fasciclin-III, strongly expressed in the polar pairs, the specialized follicle cells (FC) involved in oocyte polarity. No differences between control and *M6* ^01^ were observed with either marker through early and middle oogenesis (Fig. S2A,B).

As mentioned earlier, the *M6* ^01^ phenotype is reminiscent of defects in eggshell formation [20], [21], [24], [29], [30]. So, we next examined in *M6* ^01^ ovaries the localization of two major components of the vitelline membrane, sV17 and sV23, since *sV23* mutant eggs also collapse [31]. However, we did not detect signs of sV23 or sV17 delocalization either in *M6* ^01^ (Figs. S2C and S3B–C) or in transheterozygote *M6* ^01^/*M6* ^03^ egg chambers (Fig. S3B,C). Moreover, although it has been shown that the microvilli of the FC are involved in the biogenesis of the vitelline membrane [20], no clear differences in the structure of the FC microvilli between *M6* ^01^ and control ovaries were observed employing Cad99C as the specific marker (Fig. S2D). Finally, as alterations of various steps in yolk production are also associated with an egg collapse phenotype [32], [33] we tested the localization of the vitellogenin receptor, Yolkless, in mutant egg chambers in order to assess vitellogenin endocytosis. However, we did not detect altered Yolkless localization throughout oogenesis in *M6* ^01^ and *M6* ^01^/*M6* ^03^ mutants (Figs. S2E and S3D). Thus, further analysis will be required to define which step in eggshell assembly is affected upon reduction of *M6* levels.<

### M6 localizes to the membrane of the follicular epithelium

In an attempt to assess M6 localization we generated antibodies against M6 which failed to efficiently recognize the endogenous protein. Instead, we took advantage of an enhancer trap line carrying a promoterless GFP sequence that had been mapped to *M6*, renamed *M6* ^GFP^ (Fig. 4A, [17]). Expression of GFP-M6 in *M6* ^GFP^ ovaries was tested by RT-PCR. DNA sequencing confirmed that GFP is fused to the N-terminal of M6 isoforms derived from P1 (Fig. 4A). Although *M6* expression was slightly increased in *M6* ^GFP^ ovaries, *M6* ^GFP^ females did not show any fertility phenotype (Fig. S4A, B). Thus, immunofluorescence of *M6* ^GFP^ ovarioles was carried out to spatially and temporally localize GFP-M6.

M6-associated GFP signal was detected in the FC throughout oogenesis (Figs. 4C–F, S4C and S5). Specifically, M6 localized along the entire lateral and basal membranes but appeared more concentrated to the microvilli in the apical membrane of the FC (Fig. 4C d-d′); besides, it was detectable in the tricellular junctions of the basal membrane (Fig. 4C b′–d, d′″), and particularly in the open zone of contact (open-ZOC, Fig. 4C b″), recently implicated in cell adhesion [34], [35].

We next costained *M6* ^GFP^ ovarioles with follicular markers. Armadillo/β-catenin confirmed GFP-M6 localization to the membrane of the FC (Figs. 4C a–c and S4C). Additionally, Fasciclin III and GFP-M6 colocalized during early and mid stages (Figs. 4D and S5A), indicating that M6 is strongly expressed in both anterior and posterior polar cells, as well as in the migrating border cells. During stages 9–12 the squamous FC over the nurse cells did not show GFP-M6, implying that GFP-M6 is absent from this subset of FC. Further M6 localization to the microvilli through stages 9 to 10B was confirmed using anti-Cad99C (Figs. 4E and S5B). Interestingly, lack of co-staining with the cortex marker Yolkless [22], excluded GFP-M6 from the oocyte (Fig. 4F), which coupled with the observation that GFP-M6 is not expressed in nurse cells suggests that GFP-M6 is absent from the germline.

Based on this characterization we conclude that GFP-M6 localizes to the plasma membrane of the follicular epithelium (FE) throughout oogenesis, implying it plays a role specific to the FE.

### M6 knockdown restricted to the follicular epithelium phenocopies *M6* ^01^ ovarian phenotypes

Given that GFP-M6 is specifically expressed in the follicular epithelium we examined M6 function restricted to this tissue. To do so we downregulated *M6* expression with the GAL4/UAS system [36] using two independent *M6*-RNAi lines. Employing ubiquitous promoters, lethality became apparent during early development, when M6 localizes to the central nervous system and likely to the epithelia (Fig. S6A). Interestingly, certain FE-specific enhancer traps triggered lethality at pupal stages (Fig. S6B), supporting a role for M6 during embryogenesis and metamorphosis (Table S2).

**Figure 5:**
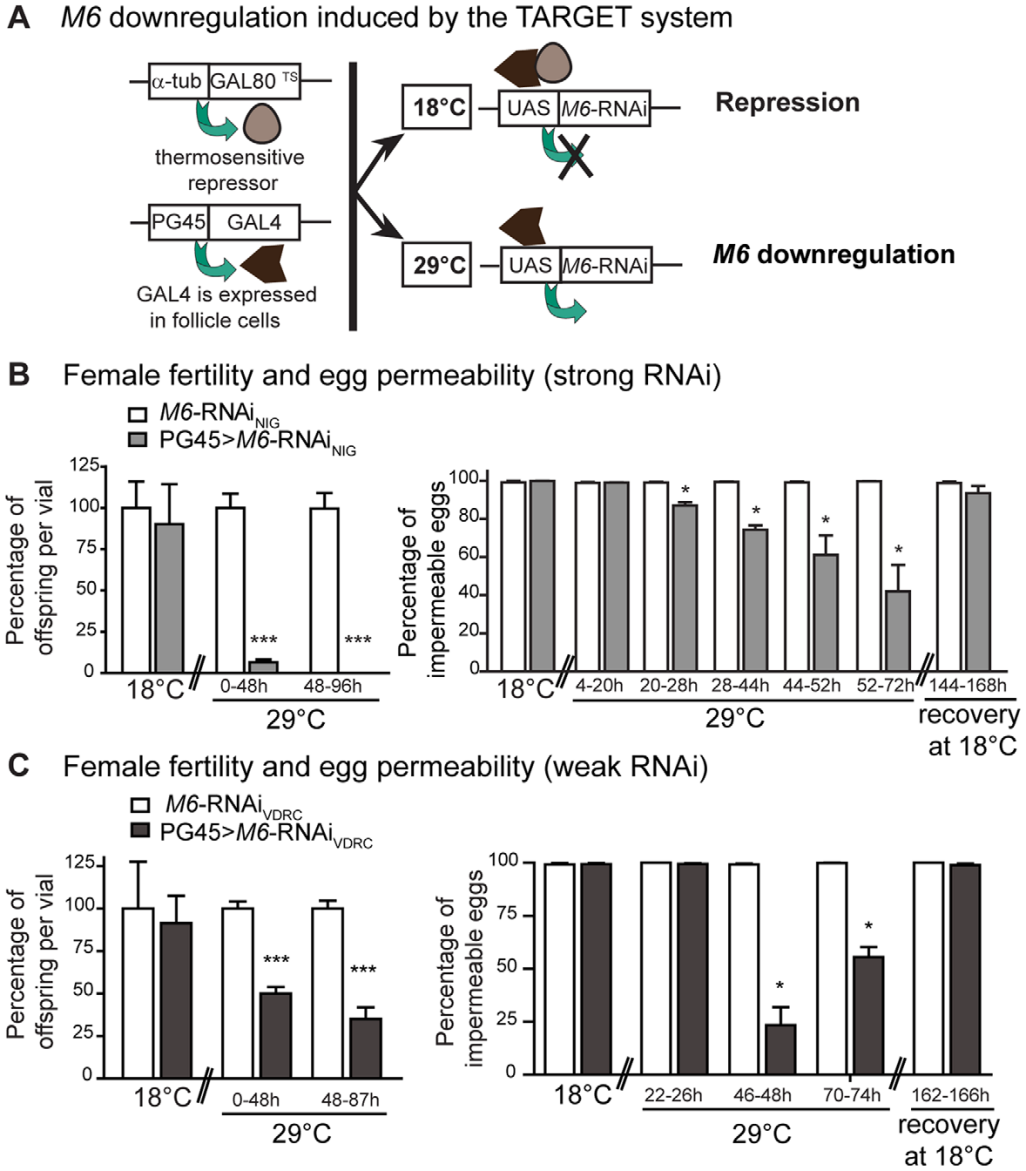
Tissue-specific *M6* downregulation induces female sterility and egg permeability. (A) Outline of the TARGET system used to induce *M6* downregulation. GAL4 activity is regulated by a temperature-sensitive allele of the GAL80 repressor, active when maintained at 18°C [18]. Upon transfer to 29°C the repression is released. Adult females were raised at 18°C and then incubated at the indicated temperature. (B–C) Female fertility and egg permeability were tested using two independent *M6*-RNAi lines, a stronger one from NIG-fly (B) and a weaker one from VDRC (C). Left panel: Female fertility was measured as in Figure 1. Females were incubated at 18°C and 29°C for 0–48 hours and 48–96 hours to induce *M6* downregulation in the FE. Values are relative to the corresponding control (white bar). Mean ± s.e.m., n = 3; Mann Whitney test between genotypes at each condition, *p*<0.001 (***). Right panel: Egg permeability was measured as in Figure 3D. After 76 hours at 29°C, flies were transferred to 18°C and allowed to recover for 144–168 h or 162–166 h, when the permeability defect was rescued. Mean ± s.e.m., n = 2; Mann Whitney test between genotypes at each condition, *p*<0.05 (*). (B) Female genotypes tested were: *w*; GAL80^TS^; *M6*-RNAi_NIG_, termed *M6*-RNAi_NIG_ (white bars) and PG45; GAL80^TS^; *M6*-RNAi_NIG_, termed PG45>*M6*-RNAi_NIG_ (grey bars). Total number of quantified eggs were 700–800 per condition per genotype, but due to oogenesis disruption (see Fig. 6C–D) only 416, 60 and 57 intact eggs were available in PG45>*M6*-RNAi_NIG_ kept at 29°C for 28–44, 44–52 and 52–72 h, respectively. (C) Female genotypes tested were: *w*; UAS-*M6*-RNAi_VDRC_; *tub*-GAL80^ts^ termed *M6*-RNAi_VDRC_ (white bars) and PG45; UAS-*M6*-RNAi_VDRC_; *tub*-GAL80^ts^ termed PG45>*M6*-RNAi_VDRC_ (dark grey bars).

Since M6 is essential throughout development we resorted to an inducible system [18] to obtain viable adult flies and restrict *M6* downregulation to adulthood. We first confirmed that the enhancer trap PG45 [24], as M6 (Fig. 4), is expressed in the FE of egg chambers throughout oogenesis (Fig. S7D). To address M6 role during the development of the FE, *M6*-RNAi was induced for several days at 29°C in PG45>*M6*-RNAi females. Controls flies included *M6*-RNAi without PG45 (*M6*-RNAi/+) maintained at 29°C and PG45>*M6*-RNAi at 18°C, a non-induced condition (Fig. 5A). We then evaluated whether FE-restricted *M6* downregulation induced the sterility phenotype displayed by *M6* ^01^ and the *M6* ^Δ01^ allelic series (Figs. 1, 2). PG45>*M6*-RNAi_NIG_ flies kept at 29°C showed a drastic reduction in female fertility compared to controls (left panel, Fig. 5B), and in contrast to the groups kept at 18°C. The driver *per se* (PG45/+) did not exhibit female sterility defects at either temperature (data not shown). Thus, M6 knockdown in the FE triggered female sterility in a tissue specific manner.

We then corroborated whether the fertility decline associated with increased egg permeability. No permeable eggs were detected in PG45>*M6*-RNAi_NIG_ and control groups at 18°C. However, a clear increase in the percentage of permeable eggs became evident in those expressing the *M6*-RNAi_NIG_ (right panel, Fig. 5B). To examine whether the reduced fertility was the result of an acute reversible effect, or triggered by a long lasting (irreversible) condition, we transferred flies kept at 29°C for 76 hours to 18°C. Strikingly, only 144–168 hours at 18°C were necessary to completely revert defects in egg permeability, most likely as a result of a normalized oogenesis in PG45>*M6*-RNAi_NIG_ females (right panel, Fig. 5B).

Next, we tested female fertility and egg permeability employing an independent RNAi line, termed *M6*-RNAi_VDRC_, which was also directed to the FE. A 50% reduction in the number of offspring in M6 knockdown flies was evidenced (left panel, Fig. 5C) and accompanied by an increase in egg permeability (right panel, Fig. 5C) that recovered upon transfer to 18°C. Thus, exploiting this inducible system we were able to turn on and off *M6* downregulation in the FE, with the concomitant induction and restoration of altered permeability. Taken together these data imply a crucial role for M6 within the FE in eggshell formation.

A closer inspection of stage 14 egg chambers from the PG45>*M6*-RNAi_VDRC_ line induced at 29°C for 72 hours showed a subtle round-shaped egg chamber phenotype (mean of the width/length ratio ± SEM; 0.382 and 0.376±0.006 for controls vs. 0.471±0.006 for *M6*-RNAi induced egg chambers, Kruskal Wallis test, *p*<0.0001, n = 50 egg chambers per genotype and condition), suggesting a failure in egg chamber elongation.

In addition, we found that *M6* downregulation driven by other follicular GAL4 lines were unable to trigger female sterility. A detailed description of the GAL4 drivers and enhancer traps used and the phenotypes displayed are summarized in Table S2 and Figure S7. Distinctive effects of specific GAL4 lines might be attributed to differences either in the strength, the spatial or the temporal GAL4 expression profiles in the FE throughout oogenesis.

### M6 knockdown in the follicular epithelium impairs oogenesis

**Figure 6:**
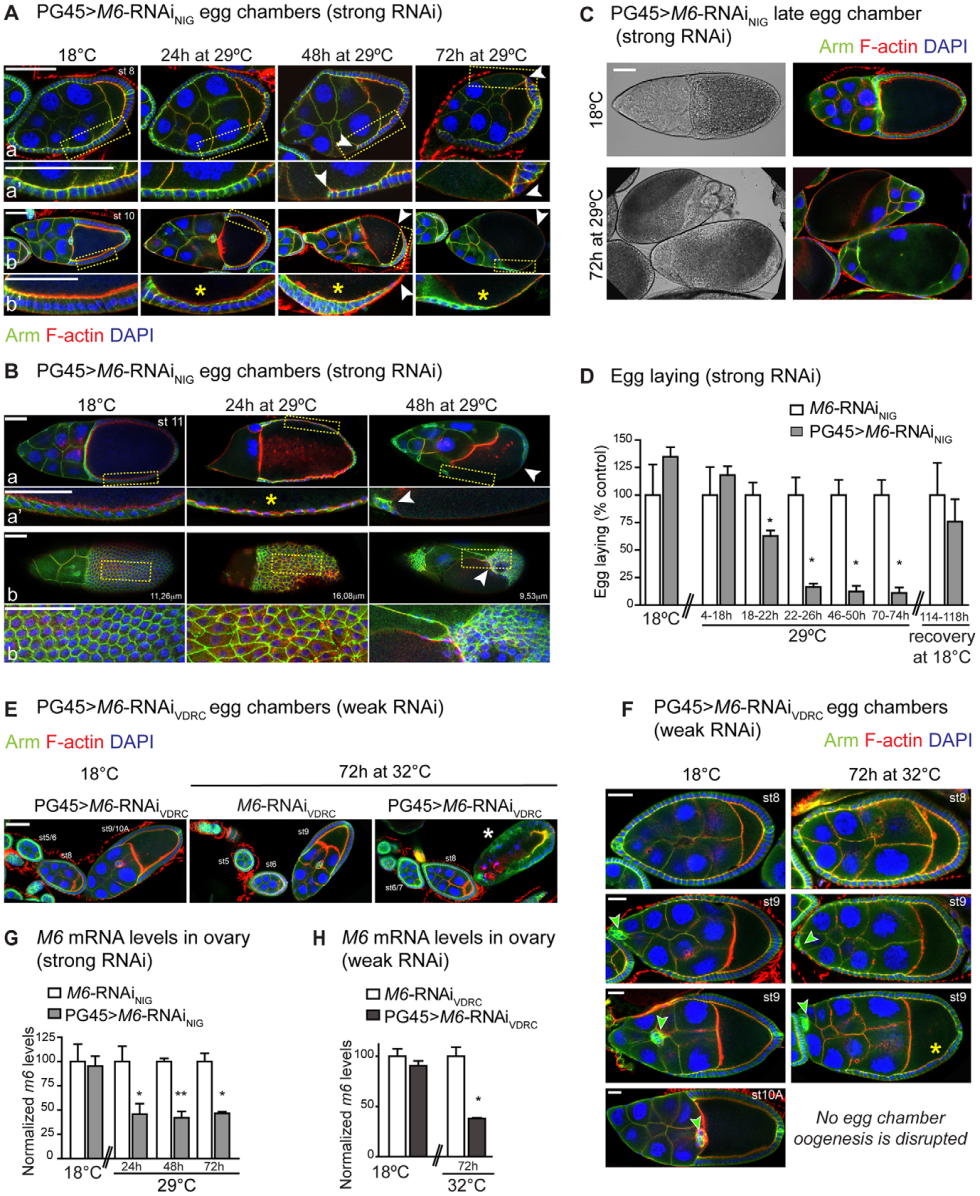
M6 knockdown in the follicular epithelium impairs oogenesis. PG45>*M6*-RNAi flies were raised at 18°C and then adults were either kept at 18°C (control) or transferred to 29°C or 32°C for 24, 48 or 72 hours to induce the *M6*-RNAi in the FE. Ovaries were dissected and stained with phalloidin (red), DAPI (blue) and Armadillo (green; A–C, E–F). Confocal images of PG45>*M6*-RNAi_NIG_ (PG45; *tub*-GAL80^ts^; UAS-*M6*-RNAi_NIG_) egg chambers corresponding to early and mid-oogenesis (A) and late oogenesis (B–C) are shown. Gaps and loss of FE organization and integrity are indicated by white arrowheads. Note defects in the FC shape (yellow asterisks, *). (B) After a 72 hour induction of *M6* knockdown, egg chambers reaching late oogenesis could not be assigned due to gross morphological defects. Two views of a st11 egg chamber incubated at 29°C for 24 hours and 48 hours are presented in (a, 0 µm) and (b, 11.26 µm). (a′–b′) Magnified views of the indicated regions. Supplementary information is presented in Fig. S8. (C) Light transmission and fluorescent images of late egg chambers incubated at 18°C (upper panel) and 29°C for 72 hours (lower panel). Note the aberrant morphology induced by *M6* downregulation. Scale bar is 50 µm. (D) Egg laying was determined as in Figure 3A. PG45>*M6*-RNAi_NIG_ (grey bars) and control females, without the PG45 driver (white bars), were incubated at 18°C and transferred to 29°C. After 76 hours at 29°C, flies were allowed to recover at 18°C for 144–168 hours and the phenotype was restored. Mean ± s.e.m., n = 2; Mann Whitney test between genotypes at each condition, *p*<0.05 (*). (E–F) Confocal images of egg chambers from PG45>*M6*-RNAi_VDRC_ (PG45; UAS-*M6*-RNAi_VDRC_; *tub*-GAL80^ts^) females incubated at 18°C or 32°C for 72 h are shown. (E) Arrested ovarioles were detected in PG45>*M6*-RNAi_VDRC_ incubated at 32°C for 72 h, which were never evidenced in controls. The white asterisk indicates an arrested st9 egg chamber. Scale bar is 50 µm. (F) No late st9 was observed in PG45>*M6*-RNAi_VDRC_ induced at 32°C for 72 h. Yellow asterisks point to defects in follicle cell shape, which are not as columnar as control, whereas green arrowheads indicate the position of polar and border cells (st9). Note that at 32°C the cluster of border and polar cells did not migrate posteriorly in late st9 egg chambers. Scale bar is 20 µm. (G–H) *M6* mRNA levels were measured in ovaries by RT-qPCR. (G) Ovaries from PG45>*M6*-RNAi_NIG_ (grey bars) and control females, without the PG45 driver (white bars), were dissected from adult females incubated at 18°C or at 29°C for 24, 48 and 72 hours. Mean ± s.e.m., n = 3; unpaired *t*-tests with Welch correction between genotypes, *p*>0.05 at 18°C; *p*<0.05 at 29°C for 24 hours and 72 hours (*); *p*<0.01 at 29°C for 48 hours (**). (H) Ovaries from PG45>*M6*-RNAi_VDRC_ (dark grey bars) and control females, without the PG45 driver (white bars), were dissected from adult females incubated at 18°C or at 32°C for 72 hours. Mean ± s.e.m., n = 3; unpaired *t*-test with Welch correction between genotypes, *p*>0.05 at 18°C, *p*<0.05 at 32°C (*).

To further analyze M6 function we performed immunohistochemistry in ovarioles from control and *M6*-RNAi_NIG_-induced females. Loss of M6 triggered a number of developmental defects such as disorganization and even total disruption of the FE as revealed by the presence of gaps (Figs. 6A,B and S8; white arrowheads). Other developmental defects included altered follicle cell shape changes revealed as the loss of columnar FC in mid-oogenesis after a 24 hour induction (Figs. 6A,B and S8B; yellow asterisk) accompanied with an impaired centripetal migration of FC at st10B (Fig. S8B–C, E; yellow arrowheads), probably leading to smaller oocytes (extending less than half of the egg chamber at stage 10; Fig. S8B–C, E; white arrows). Besides, the FC failed to flatten to accommodate the growth of the oocyte at later stages after a 48 hour induction (Figs. 6B and S8). Moreover, stretched FC were not detectable at 48 or 72 hours (Figs. 6A–B and S8). Sustained *M6* downregulation for 72 hours resulted in abnormal egg chambers that could not be staged (Fig. 6C). Gaps (defined as interruption of the FE, Figs. 6 A,B and S8A, E; white arrowheads), altered FC shape changes in late st9 and st10 (cuboidal to columnar, Figs. 6A,B and S8B; yellow asterisks) and disorganized FE in st11-12 (i.e. an alteration in fairly uniform hexagonal array, Figs. 6B and S8D) were quantitated (Table 1). Most egg chambers showed the three alterations just described. In addition to FE disorganization, abnormal Armadillo accumulation at the tricellular junctions of FE in st12-13 egg chambers was detected when the *M6*-RNAi_NIG_ was induced for 24 h at 29°C (Fig. S8D).

**Table 1:** Quantitative evaluation of the morphological defects observed in the follicular epithelium of PG45>*M6*-RNAi_NIG_ egg chambers (strong RNAi).

	FC shape^a^	Disorganized FE^b^	Gaps^c^	
Egg chambers	st9late-10	st11-12	stages <7	stages >7
Induced at 29°C	95,8% (48)	96% (50)	3,8% (106)	96,2% (106)
Control at 29°C	0% (21)	0% (17)	0% (78)	0% (74)
Non-induced	0% (21)	0% (15)	0% (51)	0% (62)

*M6* downregulation was induced as in Figs. 5 and 6. Female genotypes tested were *w*; UAS-*M6*-RNAi_NIG_; *tub*-GAL80^ts^ (termed ¨control ¨), and PG45; UAS-*M6*-RNAi_NIG_; *tub*-GAL80^ts^ either kept at 18°C ( ¨Non-induced ¨ control) or at 29°C ( ¨Induced ¨) for 24 h (a,b) or 48 h (c). Ovaries were dissected and stained to visualized morphological defects. Values correspond to the percentage of egg chambers showing the specific alteration (described in a, b or c). The number of egg chambers examined is indicated between brackets. (a) Changes in the shape of the FC, i.e. columnar as opposed to cuboidal (yellow asterisk in Fig. 6Ab′), were considered. This phenotype was analyzed in egg chambers from late st9 to 10. (b) Loss of FE organization, including a variety of polygonal shapes instead of the uniformly hexagonal array characteristic of FC (Fig. 6Bb-b′). Egg chambers from st11 to 12 were analyzed. (c) Presence of gaps within the FE (white arrowheads in Fig. 6A,B). Egg chambers from early oogenesis (st<7) and mid- to late oogenesis (st>7) were analyzed. FC, follicle cells; FE, follicular epithelium.

To quantify the extent of the defect the number of eggs laid per female was determined. A drastic reduction in the number of eggs laid by RNAi-induced females became evident (Fig. 6D). No differences were found at 18°C. As mentioned earlier (Fig. 5B), a complete rescue of the phenotype was observed several hours upon transfer to 18°C (Fig. 6D).

As female fertility in PG45>*M6*-RNAi_VDRC_ was only partially reduced (∼50%, Fig. 5C), we hypothesized that *M6*-RNAi_VDRC_ is less efficient in reducing *M6* levels, and considered a ¨weaker̈ line. To increase GAL4 activity and, concomitantly, the strength of *M6* downregulation, flies were transferred to 32°C for 72 hours, which triggered a clear disruption in oogenesis (Fig. 6E; white asterisk). In contrast, controls incubated at 32°C did not show any defect (Fig. 6E). At 32°C no egg chambers in stages from 9 to 13 could be identified (Fig. 6F). In addition, both polar and border cells in stage 9 did not migrate and remained localized to the anterior region (see Fig. 6F, green arrowheads).

To confirm that oogenesis disruption was effectively induced by *M6* downregulation, *M6* mRNA levels were assessed by RT-qPCR. As expected, a clear reduction in *M6* levels was observed in both *M6*-RNAi-induced ovaries (Fig. 6G, H), down to ∼46% in PG45>*M6*-RNAi_NIG_ induced for 72 hours at 29°C (Fig. 6G) and ∼38% in PG45>*M6*-RNAi_VDRC_ induced for 72 hours at 32°C (Fig. 6H). In contrast, no differences were observed in *M6* mRNA levels between PG45>*M6*-RNAi and control ovaries at 18°C.

In sum, acute downregulation of *M6* levels in the FE gives rise to a number of defects leading to disruption in oogenesis.

## Discussion

We identified M6 as a protein involved in egg chamber development that when expressed at low levels gives rise to female sterility in *Drosophila*. M6 is expressed in different populations of follicle cells throughout oogenesis, and localizes to the plasma membrane. Such subcellular distribution was predicted from the degree of conservation with other PLP family proteins [9], [10], [15], [16]. The mammalian ortholog, M6a, is also expressed in non-neural tissues [1], [8], where it appears particularly concentrated in the apical surface along a highly dense array of microvilli [1]. M6 distribution to membrane protrusions of epithelial cells is reminiscent of that reported for mammalian M6a. M6 is also expressed in the fly embryo and is essential at later stages in development, suggesting it plays an additional role during embryogenesis and metamorphosis. Interestingly, downregulated expression of both proteins, M6 in *Drosophila* and M6a in mammals, correlates with pathologies: female sterility and chronic stress, respectively. Notably, in both cases, a cell remodeling process is involved.

Using different genetic strategies such as the insertional mutants and M6 knockdown in the FE we demonstrated that M6 plays an essential role during egg chamber development. Interestingly, in addition to the egg permeability phenotype displayed by *M6* ^01^, tissue-specific knockdown disrupted oogenesis. While the P-element in *M6* ^01^ is inserted in the first exon of promoter 1 (P1, Fig. 1A), and thus potentially affects a subset of *M6* splice variants, both *M6*-RNAi sequences were targeted to all *M6* mRNAs. Remarkably, RNAi lines obtained from different collections (NIG and VDRC) led to similar phenotypes, indicating that egg permeability, altered follicular epithelium integrity and arrested egg chamber development were indeed the result of downregulated M6 expression in the FC. However, despite *M6* downregulation in both *M6* ^01^ and *M6*-RNAi treated ovaries reached similar *M6* mRNA levels (40–45%), the ovarian phenotypes displayed by *M6*-RNAi lines were stronger than *M6* ^01^, implying a nonlinear relationship between the two. Moreover, *M6* locus gives rise to different M6 versions as a result of the use of alternative promoters and alternative mRNA splicing (Zappia *et al.*, unpublished results), underlying the complexity of this locus. We can envision at least two explanations to reconcile this apparent discrepancy. On the one hand, although both *M6*-RNAi putatively target all isoforms, their expression was limited to the PG45 domain. This might not completely describe the *M6* endogenous pattern evidenced by the enhancer trap GFP-M6, which in turn reflects the pattern of isoforms driven by P1 (and not P2). Thus, it is possible that additional tissues (such as the germline) could contribute the remaining *M6* levels detected by qPCR in whole ovarian extracts. On the other hand, the insertion in *M6* ^01^ could spare specific isoforms (such as those driven by P2), which could be differentially expressed in ovaries and contribute to the detected *M6* levels. This topic is the focus of intense analysis and will be reported elsewhere (Zappia *et al.*, unpublished observations).

Several evidences point to a temporal window for M6 requirement, which coincides with the time the FE undergoes tissue remodeling [37], [38]. Using two alternative enhancer trap lines to drive M6 knockdown only the one that is active from stage 7 onwards triggered the sterility phenotype. In addition, morphological defects induced with *M6*-RNAi lines were first observed at stage 9. Thus, while oogenesis apparently proceeded relatively normally from germarium through stage 8, morphological defects triggered by downregulated *M6* expression became detectable at later stages.

During the second half of oogenesis the FC have two main roles: the communication with the oocyte that is essential for patterning the egg chamber and early embryo, as well as the formation of the eggshell. The latter is characterized by a sequence of migrations and cell shape changes involving subpopulations of FC that cover the oocyte and synthesize specialized eggshell structures. During mid-oogenesis the epithelial FC cease proliferation (stage 7), and form a cuboidal epithelium surrounding the whole egg chamber (stage 8) [37]. In stage 9 the FC monolayer undergoes cell shape changes, concurrent with border cell migration [39]. In contrast, upon tissue-specific M6 knockdown the follicular monolayer was disorganized, did not achieve a columnar morphology and stayed cuboidal even at stages when elongation should be occurring.

Later on, as a result of both mechanical stretching and movement, the FC flatten through the expansion of their basal and apical membrane domains, which becomes evident at stage 10B. The membrane required for the expansion of the apical side (facing the oocyte) is probably stored in microvilli. However, there is no membrane reservoir in the basal one; consequently, a portion of the lateral membrane previously engaged in cell-cell interactions called zone of contact (ZOC) is converted into basal membrane (open ZOC) and faces the basal extracellular matrix. Such plasma membrane remodeling strengthens adhesion and ensures epithelial integrity at the next stages of development [34], [35]. Since GFP-M6 localizes at the ZOC and at the microvilli of FC, and its downregulation induces epithelial monolayer disruption, M6 may contribute to membrane remodeling processes such as cell-cell adhesion, changes in cell shape, signaling and membrane trafficking, and thus be essential for maintaining the FE integrity, although additional experiments are required to confirm this hypothesis. Work from different laboratories has focused on the maintenance of the FE integrity during egg chamber development and showed the requirement of the cadherin–catenin complex [40], the serine/threonine kinase dPak [41], and integrins [42]. Additionally, *M6* downregulation in FC triggered a dumpless-like phenotype. Although it usually implies structural defects in ring canals or actin-organization, it could also arise as a result of altered centripetal migration as observed at st10 late (Fig. S8C) and suggested by Dobbens and col. [39], pointing out that cell-cell communication between the follicular epithelium and the germ cells coordinate FC morphogenesis.

During late oogenesis, the structural components of the eggshell are secreted by the FC surrounding the oocyte in a precisely regulated temporal and spatial manner. In normal eggs the vitelline membrane and the wax layer prevent dehydration. Abnormalities in the deposition of either one of these structures or, alternatively, a failure to completely cover the anterior end of the oocyte upon migration of the anterior FC might cause eggs to loose water and show a collapsed phenotype [43]. M6 expression in FC secreting the eggshell material is also consistent with a role in this process.

In *Drosophila* gut and wing epithelia, integrins are implicated in the maintenance of columnar morphology [44], [45]. Integrins could be playing a similar role in the FE overlying the oocyte. Indeed, distinct integrins alpha chains showed both temporal and spatial pattern expression differences in FC during mid- and late oogenesis [46] and newly synthesized basolaterally localized integrins are involved in the transition from columnar to cuboidal morphology in the follicular epithelium [34]. Our data suggests that both processes, FC shape changes and vitelline membrane assembly, are closely related and dependent on M6 function.

Mutations that cause spherical egg phenotype are known to disrupt proteins mediating interactions between the actin cytoskeleton and the extracellular matrix [47], [48], [49], [50]. Since the round egg chamber phenotype triggered by *M6* downregulation is subtle, additional studies are needed to investigate whether M6 plays a role during the planar-polarized orientation of basal F-actin bundles in the follicular epithelium.

The failure in the maintenance of epithelial architecture induced by *M6* downregulation may be due to defects in cell adhesion leading to alterations in cell shape, which in turn disrupt the epithelial organization. Preliminary studies supporting this hypothesis suggest altered distribution of DE-cadherin at the tricellular junctions of *M6* ^01^ late stages egg chambers (Zappia and col., unpublished results). A potential role of M6 in cell adhesion is further supported by the fact that PLP was proposed to act as an adhesion molecule [13] and to form a complex with integrins in oligodendrocytes [51]. In addition, mammalian M6a localization in primary cultures of hippocampal neurons [5] and other epithelia [1], [8], and PLP/DM20 in HeLa cells [52], suggest that a common feature in the PLP family is their ability to associate with membrane structures involved in movement and growth [12]. In summary, we have uncovered an essential role of M6 in *Drosophila* oogenesis, which is particularly relevant in light of the conservation of this protein between flies and mammals.

## Supporting Information

Figure S1 **The P element insertion in** ***M6*** **^01^ does not affect expression in the nearby CG33214 locus.** The upstream locus contiguous to *M6*, CG33214, which transcription initiation is only 384 pb away from the insertion site in *M6* ^01^, was not affected by the P element insertion. Expression of CG33214 was measured in ovaries from wild type, *M6* ^01^ and *M6* ^Δ01-rev^ (imperfect excision) by RT-qPCR and normalized to *Rp49*. Mean ± s.e.m, n = 3. Statistical analysis included One Way Anova (p>0.05).
(TIF)

Figure S2 ***M6*** **^01^ ovarioles show neither gross morphological defects nor altered localization of vitelline membrane biosynthesis markers.** Immunofluorescence of wild type (*w*, upper panel) and *M6* ^01^ (lower panel) egg chambers with antibodies directed to Armadillo (A), Fasciclin III (green, B), sv23 (green, C), Cad99C (green, D) and Yolkless (green, E). (B–E) Egg chambers costained with phallloidin to visualize F-actin (red). Scale bar is 50 µm, unless otherwise indicated.
(TIF)

Figure S3 ***M6*** **^01^ and** ***M6*** **^01^/** ***M6*** **^03^ egg chambers do not show altered localization of vitelline membrane proteins.** (A) Eggs laid by heterozygote *M6* ^01^/+ and *M6* ^03^/+ and transheterozygote *M6* ^01^/*M6* ^03^ females were collected and incubated with the neutral red dye. All (100%) *M6* ^01^/*M6* ^03^ mutant eggs were permeable, which is taken as an indication of an abnormal vitelline membrane. (B–D) Immunofluorescence of wild type (*w*, left panel), *M6* ^01^ (middle panel) and *M6* ^01^/*M6* ^03^ (right panel) sectioned egg chambers with antibodies directed to sV23 (green, B), sV17 (green, C) and Yolkless (green, D). Numbers indicate egg chambers stages. Nuclei are visualized in red (DAPI). Projection of z-stacks are shown in (Cc.) and (Da,b). *M6* mutant egg chambers displayed a continuous line of the vitelline membrane proteins (sV17 and sV23 staining), and no relocalization into the oocyte. Scale bar is 50 µm. Oo, oocyte; Fc, follicle.
(TIF)

Figure S4 **GFP-M6 colocalizes with Armadillo in the follicular epithelium.** (A) Female fertility assessment of *M6* ^GFP^ flies. Female fertility was measured as the number of offspring per vial obtained when crossed to wild type males. Mean ± s.e.m, n = 3; unpaired *t*-test with Welch correction, *p*<0.05. (B) *M6* mRNA levels in ovaries was measured in wild type (*w*) and *M6* ^GFP^ flies by RT-qPCR and normalized to *Rp49* or *gapdh*. Statistical analysis included an unpaired *t*-test with Welch correction, *p*>0.05; mean ± s.e.m, n = 3. (C) Armadillo/β-catenin labels the membrane of follicle and nurse (to a lower extent) cells (magenta). GFP-M6 is localized to the membrane of the follicle cells throughout oogenesis in *M6* ^GFP^ (green). Colocalization is shown on the right panel. Representative egg chambers are shown. F-actin was labeled with phalloin (red) and nuclei with DAPI (blue, left panels). Scale bar is 20 µm, unless otherwise indicated.
(TIF)

Figure S5 **GFP-M6 colocalizes with Fasciclin and Cad99C.** GFP-M6 (green) localizes to polar (also labeled with Fasciclin III, in magenta) and border cells (A) and to microvilli of the follicle cells labeled with anti-Cad99C antibodies (B, in magenta) in *M6* ^GFP^. Colocalization is shown on the right panel. Representative egg chambers are shown. F-actin was labeled with phalloidin (red) and nuclei with DAPI (blue, left panels). Scale bar is 20 µm.
(TIF)

Figure S6 **M6 requirement during development.** (A) GFP-M6 is expressed in the embryo. Immunofluorescence images at different depths from the same *M6* ^GFP^ embryo are presented in the upper and lower panels. Fasciclin II [red; anti-FasII MAb 1D4 (1∶5; DSHB)] labels longitudinal axon fascicles in the nervous system. GFP-M6 (green) is expressed in the longitudinal fascicles as shown by the colocalization (right panel), as well as in other tissues such as the epithelium (lower panel). Therefore, M6 knockdown in the embryo might be responsible for early lethality (see Table S2 for details). Scale bar is 50 µm. (B) When employing various enhancer traps reported to be expressed in the follicle cells to induce *M6*-RNAi, such as c204 and T155 (Bateman et al., 2001), an arrested pupae development was observed, suggesting that in addition to driving expression in follicle cells, those GAL4 drivers are also expressed at an unspecified tissue during larval/pupae formation. Thus, M6 deprivation during larval or pupae development stages abrogates metamorphosis (see also Table S2). Images of day 10 (upper panel) and 15 (lower panel) of *Drosophila* development at 25°C from flies bearing the enhancer traps c204 and the strong allele of *M6*-RNAi (C204>*M6*-RNAi_NIG_) and control flies (*M6*-RNAi_NIG_ with no driver). Pupae arrest was detected during metamorphosis while control flies developed normally. Similar results were observed using T155. Scale bar is 2 mm.
(TIF)

Figure S7 **Follicular GAL4 drivers used to downregulate M6 in FC.** Representative images of immunofluorescence of (A) 55B>CD8GFP, (B) da.G32>CD8GFP, (C) slbo>CD8GFP, (D) PG45>CD8GFP, (E) c355>CD8GFP, (F) c204>CD8GFP, (G) T155>CD8GFP, (H) c329b>CD8GFP and (I) tubP>CD8GFP ovarioles. GAL4 expression is revealed by CD8-GFP labels (green) in follicle cells. Nuclei were stained with propidium iodide or DAPI (red). PG45 expressed GAL4 in all follicle cells throughout oogenesis (D). Note GAL4 expression in subpopulation of follicle cells with different drivers (A,B,C and H). Interestingly, those follicular drivers (55B, da.Gal4, slbo.Gal4 and c329b) did not trigger any female sterility in the context of *M6*-RNAi, whereas PG45 did (see also Table S2). GAL4 is expressed in all follicle cells from st7 onwards (c355, (E)) and from st8/9 onwards (c204, (F)). The specific spatial or temporal expression profiles or GAL4 expression strength may account for the differences observed in female sterility phenotype when crossed to the *M6*-RNAi (see also Table S2). Representative egg chambers are shown. Scale bar is 50 µm.
(TIF)

Figure S8 ***M6*** **downregulation in follicle cells induces a variety of defects in mid- and late oogenesis.** Confocal images of representative egg chambers at different stages of oogenesis. Ovaries were dissected and stained with phalloidin (red), DAPI (blue) and Armadillo (green). PG45>*M6*-RNAi_NIG_ (PG45; *tub*-GAL80^ts^; UAS-*M6*-RNAi_NIG_) flies were raised at 18°C and then adult females were transferred to 29°C for 24, 48 or 72 hours to induce *M6* interference in the follicular epithelium or kept at 18°C as the non-induced control. Control females, *M6*-RNAi_NIG_ (without PG45), shifted to 29°C for 72 hours were undistinguishable from non-induced controls (data not shown). Egg chambers corresponding to stages 4–7 and 9 (A), late stage 9, stage 10A and 10B (B), stages 10 and 11 (C), stages 12 and 13 (D) and stage 12 (E) are shown. (A) No morphological defects were observed at early stages of oogenesis. The presence of gaps in the follicular epithelium after 48 h of *M6*-RNAi induction became detectable from stage 8 onwards (indicated by white arrowheads), and from stage 7 onwards after 72 h of induction. Note defects in the FC shape. (B) The first defects observed after a 24 hour induction involved follicle cell morphology in egg chambers from mid-oogenesis, including a deficit in the thickness gradient of the posterior follicle cells in late stage 9 along with a failure in the columnar shape of the follicle cells in egg chambers reaching stage 10A. Defects in the overall shape of the follicle cells are indicated by yellow asterisks. Magnified views of the indicated areas are shown at the bottom of each egg chamber. White arrows highlight morphological hallmarks of the dumpless-like phenotype in st10B (i.e., disproportioned oocyte to nurse cell cellular size). (C) After a 24 hour induction, defects in centripetal migration were detected in stage 10–11 egg chambers as indicated by yellow arrowheads. This alteration might cause the dumpless-like phenotype(white arrow). (D) Disorganization of the follicular epithelium induced in stage 11–13 egg chambers after 24 hours associated with abnormal Armadillo accumulation at the tricellular junctions. (E) At later stages of oogenesis, such as stage 12, gaps were evidenced after 48 h of induction (indicated by white arrowheads). The dumpless-like phenotype (white arrow) is detected likely as a result of altered centripetal FC migration (yellow arrowhead). Scale bar is 50 µm.
(TIF)

Table S1 **Primer sequences used in PCR.** To characterize the *M6* allelic series, PCR reactions on genomic DNA obtained from homozygous flies were carried out with primers directed to the 5′UTR of *M6A* flanking the original P element insertion. The E-GFP fusion to M6 was assessed by a Hemi-Nested PCR experiment employing cDNA from *M6* ^GFP^ ovaries. Two rounds of PCR were performed in order to amplify isoforms with low representation. A second PCR experiment was carried out employing different pairs of primers. *M6* mRNA isoforms tagged to the C-terminus of E-GFP were determined by sequence alignments (KAlign algorithm; www.ebi.ac.uk). The sequences of the expressed GFP::M6 proteins were predicted *in silico*. Primer sequences used in qPCR were designed using Primer Express 3.0 software (Applied Biosystems) and targeted to the 3′UTR of all *M6* mRNA isoforms, glyceraldehyde phosphate dehydrogenase (*gapdh*) and ribosomal protein 49 (*Rp49*). To confirm *M6* levels in the *M6* allelic series a second pair of primers for M6 3′UTR was used.
(DOC)

Table S2 **M6 knockdown triggers lethality during development and sterility in adult females.** The table summarizes both the developmental arrest phenotype (middle column) and the female sterility phenotype (middle column) induced by *M6*-RNAi. Different GAL4 drivers and enhancer trap lines (left column) were used to express two independent UAS-*M6-*RNAi lines from the NIG-fly (strong allele) and VDRC (weak allele) stocks centers. Interference was amplified including UAS-*Dicer2* (from VDRC). Early development arrest is triggered upon *M6* downregulation. When pleitropic GAL4 drivers such as tubP-GAL4 [24], or follicular-specific GAL4 drivers such as the enhancer trap GAL4-daG32 [20] and PG45 [24], which are also expressed in the embryo, were used to trigger M6 interference, early lethality was observed. In every genetic combination (GAL4 driver>*M6*-RNAi) lethality became apparent at early stages during development, suggesting an essential role for M6 early on. This was also evidenced by the lethality of the *M6* ^03^ mutant and a number of *M6* alleles generated by P element excision of the *M6* ^01^ mutant (data not shown). Some of the drivers required the addition of an allele of UAS-Dicer2 to trigger the same interference with both RNAi stocks (data not shown). Only complete female sterility was scored as female sterility. Although differences in the strength of the GAL4 drivers cannot be ruled out, *M6* downregulation induced female sterility only when *M6*-RNAi was expressed in all follicle cells from st7 onwards (PG45, c355 and tubP-GAL4). Follicular GAL4 drivers were crossed to UAS-CD8GFP to check GAL4 expression in follicle cells (right column and Fig S7). Discrepancies from the literature were detected. (*) employing tub-GAL80^TS^ to repress *M6*-RNAi through development at 18°C. Then, *M6*-RNAi was induced in young adult females at 29°C. (**) tested with *M6*-RNAi_NIG_. (***) tested with *M6*-RNAi_VDRC_ FC for follicle cells.
(DOC)

## References

[1] Lagenaur C, Kunemund V, Fischer G, Fushiki S, Schachner M (1992). Monoclonal M6 antibody interferes with neurite extension of cultured neurons.. J Neurobiol.

[2] Yan Y, Narayanan V, Lagenaur C (1996). Expression of members of the proteolipid protein gene family in the developing murine central nervous system.. J Comp Neurol.

[3] Alfonso J, Aguero F, Sanchez DO, Flugge G, Fuchs E (2004). Gene expression analysis in the hippocampal formation of tree shrews chronically treated with cortisol.. J Neurosci Res.

[4] Alfonso J, Frick LR, Silberman DM, Palumbo ML, Genaro AM (2006). Regulation of hippocampal gene expression is conserved in two species subjected to different stressors and antidepressant treatments.. Biol Psychiatry.

[5] Alfonso J, Fernandez ME, Cooper B, Flugge G, Frasch AC (2005). The stress-regulated protein M6a is a key modulator for neurite outgrowth and filopodium/spine formation.. Proc Natl Acad Sci U S A.

[6] Brocco MA, Fernandez ME, Frasch AC (2010). Filopodial protrusions induced by glycoprotein M6a exhibit high motility and aids synapse formation.. Eur J Neurosci.

[7] Michibata H, Okuno T, Konishi N, Kyono K, Wakimoto K (2009). Human GPM6A is associated with differentiation and neuronal migration of neurons derived from human embryonic stem cells.. Stem Cells Dev.

[8] Baumrind NL, Parkinson D, Wayne DB, Heuser JE, Pearlman AL (1992). EMA: a developmentally regulated cell-surface glycoprotein of CNS neurons that is concentrated at the leading edge of growth cones.. Dev Dyn.

[9] Mobius W, Patzig J, Nave KA, Werner HB (2008). Phylogeny of proteolipid proteins: divergence, constraints, and the evolution of novel functions in myelination and neuroprotection.. Neuron Glia Biol.

[10] Schweitzer J, Becker T, Schachner M, Nave KA, Werner H (2006). Evolution of myelin proteolipid proteins: gene duplication in teleosts and expression pattern divergence.. Mol Cell Neurosci.

[11] Fernandez ME, Alfonso J, Brocco MA, Frasch AC (2010). Conserved cellular function and stress-mediated regulation among members of the proteolipid protein family.. J Neurosci Res.

[12] Kalwy SA, Smith R, Kidd GJ (1997). Myelin proteolipid protein expressed in COS-1 cells is targeted to actin-associated surfaces.. J Neurosci Res.

[13] Kitagawa K, Sinoway MP, Yang C, Gould RM, Colman DR (1993). A proteolipid protein gene family: expression in sharks and rays and possible evolution from an ancestral gene encoding a pore-forming polypeptide.. Neuron.

[14] Mukobata S, Hibino T, Sugiyama A, Urano Y, Inatomi A (2002). M6a acts as a nerve growth factor-gated Ca(2+) channel in neuronal differentiation.. Biochem Biophys Res Commun.

[15] Stecca B, Southwood CM, Gragerov A, Kelley KA, Friedrich VL (2000). The evolution of lipophilin genes from invertebrates to tetrapods: DM-20 cannot replace proteolipid protein in CNS myelin.. J Neurosci.

[16] Werner H, Dimou L, Klugmann M, Pfeiffer S, Nave KA (2001). Multiple splice isoforms of proteolipid M6B in neurons and oligodendrocytes.. Mol Cell Neurosci.

[17] Buszczak M, Paterno S, Lighthouse D, Bachman J, Planck J (2007). The carnegie protein trap library: a versatile tool for Drosophila developmental studies.. Genetics.

[18] McGuire SE, Mao Z, Davis RL (2004). Spatiotemporal gene expression targeting with the TARGET and gene-switch systems in Drosophila.. Sci STKE.

[19] Howlader G, Paranjpe DA, Sharma VK (2006). Non-ventral lateral neuron-based, non-PDF-mediated clocks control circadian egg-laying rhythm in Drosophila melanogaster.. J Biol Rhythms.

[20] D'Alterio C, Tran DD, Yeung MW, Hwang MS, Li MA (2005). Drosophila melanogaster Cad99C, the orthologue of human Usher cadherin PCDH15, regulates the length of microvilli.. J Cell Biol.

[21] LeMosy EK, Hashimoto C (2000). The nudel protease of Drosophila is required for eggshell biogenesis in addition to embryonic patterning.. Dev Biol.

[22] Schonbaum CP, Perrino JJ, Mahowald AP (2000). Regulation of the vitellogenin receptor during Drosophila melanogaster oogenesis.. Mol Biol Cell.

[23] Pascucci T, Perrino J, Mahowald AP, Waring GL (1996). Eggshell assembly in Drosophila: processing and localization of vitelline membrane and chorion proteins.. Dev Biol.

[24] Elalayli M, Hall JD, Fakhouri M, Neiswender H, Ellison TT (2008). Palisade is required in the Drosophila ovary for assembly and function of the protective vitelline membrane.. Dev Biol.

[25] LeMosy EK, Kemler D, Hashimoto C (1998). Role of Nudel protease activation in triggering dorsoventral polarization of the Drosophila embryo.. Development.

[26] Biosystems A (2004). Guide to Performing Relative Quantitation of Gene Expression Using Real-Time Quantitative PCR..

[27] Pfaffl MW (2001). A new mathematical model for relative quantification in real-time RT-PCR.. Nucleic Acids Res.

[28] Peifer M, Orsulic S, Sweeton D, Wieschaus E (1993). A role for the Drosophila segment polarity gene armadillo in cell adhesion and cytoskeletal integrity during oogenesis.. Development.

[29] Savant SS, Waring GL (1989). Molecular analysis and rescue of a vitelline membrane mutant in Drosophila.. Dev Biol.

[30] Schlichting K, Wilsch-Brauninger M, Demontis F, Dahmann C (2006). Cadherin Cad99C is required for normal microvilli morphology in Drosophila follicle cells.. J Cell Sci.

[31] Manogaran A, Waring GL (2004). The N-terminal prodomain of sV23 is essential for the assembly of a functional vitelline membrane network in Drosophila.. Dev Biol.

[32] Williams JL, Saunders RD, Bownes M, Scott A (1987). Identification of a female-sterile mutation affecting yolk protein 2 in Drosophila melanogaster.. Mol Gen Genet.

[33] DiMario PJ, Mahowald AP (1987). Female sterile (1) yolkless: a recessive female sterile mutation in Drosophila melanogaster with depressed numbers of coated pits and coated vesicles within the developing oocytes.. J Cell Biol.

[34] Schotman H, Karhinen L, Rabouille C (2008). dGRASP-mediated noncanonical integrin secretion is required for Drosophila epithelial remodeling.. Dev Cell.

[35] Schotman H, Karhinen L, Rabouille C (2009). Integrins mediate their unconventional, mechanical-stress-induced secretion via RhoA and PINCH in Drosophila.. J Cell Sci.

[36] Brand AH, Perrimon N (1993). Targeted gene expression as a means of altering cell fates and generating dominant phenotypes.. Development.

[37] Horne-Badovinac S, Bilder D (2005). Mass transit: epithelial morphogenesis in the Drosophila egg chamber.. Dev Dyn.

[38] Wu X, Tanwar PS, Raftery LA (2008). Drosophila follicle cells: morphogenesis in an eggshell.. Semin Cell Dev Biol.

[39] Dobens LL, Raftery LA (2000). Integration of epithelial patterning and morphogenesis in Drosophila ovarian follicle cells.. Dev Dyn.

[40] Tanentzapf G, Smith C, McGlade J, Tepass U (2000). Apical, lateral, and basal polarization cues contribute to the development of the follicular epithelium during Drosophila oogenesis.. J Cell Biol.

[41] Conder R, Yu H, Zahedi B, Harden N (2007). The serine/threonine kinase dPak is required for polarized assembly of F-actin bundles and apical-basal polarity in the Drosophila follicular epithelium.. Dev Biol.

[42] Fernandez-Minan A, Martin-Bermudo MD, Gonzalez-Reyes A (2007). Integrin signaling regulates spindle orientation in Drosophila to preserve the follicular-epithelium monolayer.. Curr Biol.

[43] Schupbach T, Wieschaus E (1991). Female sterile mutations on the second chromosome of Drosophila melanogaster. II. Mutations blocking oogenesis or altering egg morphology.. Genetics.

[44] Newman SM, Wright TR (1981). A histological and ultrastructural analysis of developmental defects produced by the mutation, lethal(1)myospheroid, in Drosophila melanogaster.. Dev Biol.

[45] Dominguez-Gimenez P, Brown NH, Martin-Bermudo MD (2007). Integrin-ECM interactions regulate the changes in cell shape driving the morphogenesis of the Drosophila wing epithelium.. J Cell Sci.

[46] Dinkins MB, Fratto VM, Lemosy EK (2008). Integrin alpha chains exhibit distinct temporal and spatial localization patterns in epithelial cells of the Drosophila ovary.. Dev Dyn.

[47] Bateman J, Reddy RS, Saito H, Van Vactor D (2001). The receptor tyrosine phosphatase Dlar and integrins organize actin filaments in the Drosophila follicular epithelium.. Curr Biol.

[48] Deng WM, Schneider M, Frock R, Castillejo-Lopez C, Gaman EA (2003). Dystroglycan is required for polarizing the epithelial cells and the oocyte in Drosophila.. Development.

[49] Frydman HM, Spradling AC (2001). The receptor-like tyrosine phosphatase lar is required for epithelial planar polarity and for axis determination within drosophila ovarian follicles.. Development.

[50] Gutzeit HO, Eberhardt W, Gratwohl E (1991). Laminin and basement membrane-associated microfilaments in wild-type and mutant Drosophila ovarian follicles.. J Cell Sci.

[51] Gudz TI, Schneider TE, Haas TA, Macklin WB (2002). Myelin proteolipid protein forms a complex with integrins and may participate in integrin receptor signaling in oligodendrocytes.. J Neurosci.

[52] Sinoway MP, Kitagawa K, Timsit S, Hashim GA, Colman DR (1994). Proteolipid protein interactions in transfectants: implications for myelin assembly.. J Neurosci Res.

[53] Drummond-Barbosa D, Spradling AC (2001). Stem cells and their progeny respond to nutritional changes during Drosophila oogenesis.. Dev Biol.

