# Supplementary figures and images for: M6 Membrane Protein Plays an Essential Role in Drosophila Oogenesis
Source: PLoS One. 2011 May 16;6(5):e19715. doi: 10.1371/journal.pone.0019715 (PMC3095610; doi:10.1371/journal.pone.0019715)

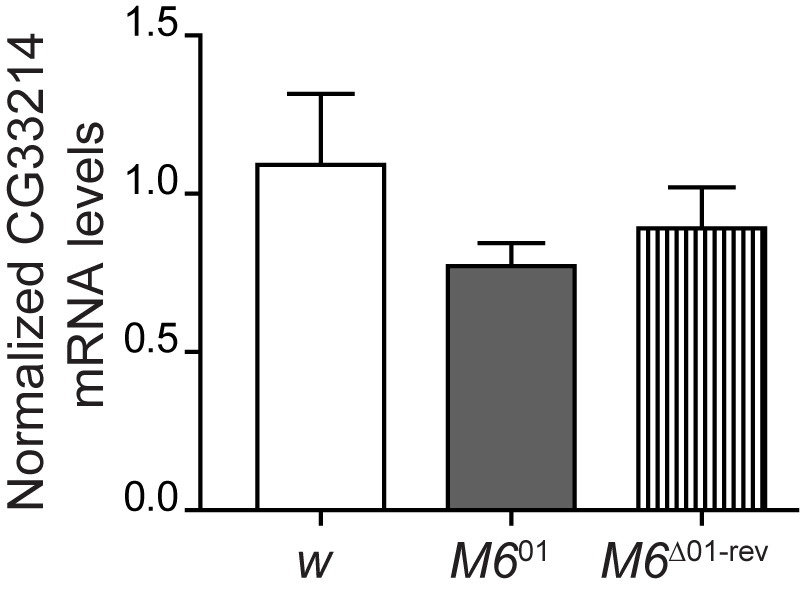

Supplement: Figure S1 — The P element insertion in M6 01 does not affect expression in the nearby CG33214 locus. The upstream locus contiguous to M6, CG33214, which transcription initiation is only 384 pb away from the insertion site in M6 01, was not affected by the P element insertion. Expression of CG33214 was measured in ovaries from wild type, M6 01 and M6 Δ01-rev (imperfect excision) by RT-qPCR and normalized to Rp49. Mean ± s.e.m, n = 3. Statistical analysis included One Way Anova (p>0.05). (TIF) [file pone.0019715.s001.tif]

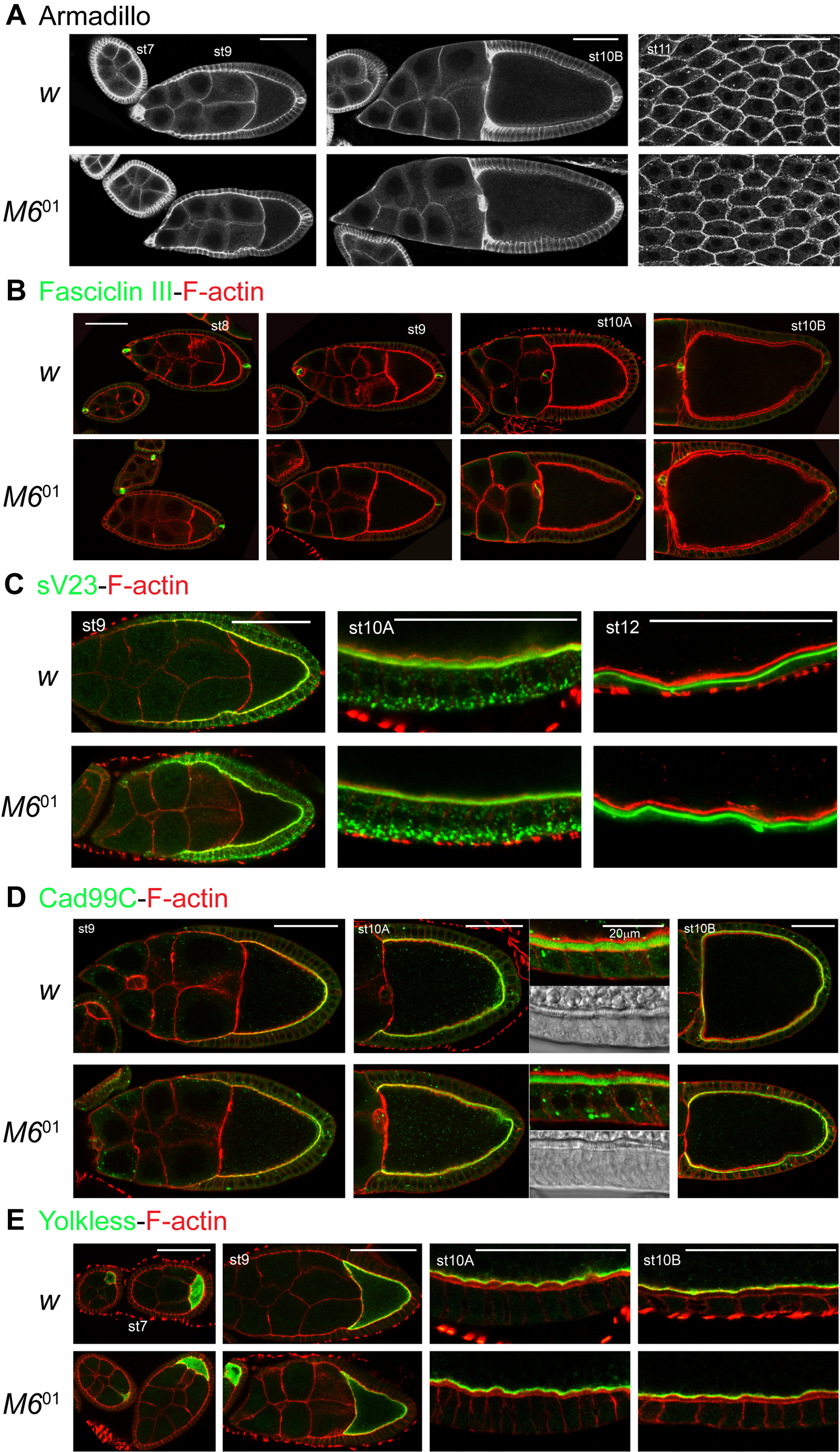

Supplement: Figure S2 — M6 01 ovarioles show neither gross morphological defects nor altered localization of vitelline membrane biosynthesis markers. Immunofluorescence of wild type (w, upper panel) and M6 01 (lower panel) egg chambers with antibodies directed to Armadillo (A), Fasciclin III (green, B), sv23 (green, C), Cad99C (green, D) and Yolkless (green, E). (B–E) Egg chambers costained with phallloidin to visualize F-actin (red). Scale bar is 50 µm, unless otherwise indicated. (TIF) [file pone.0019715.s002.tif]

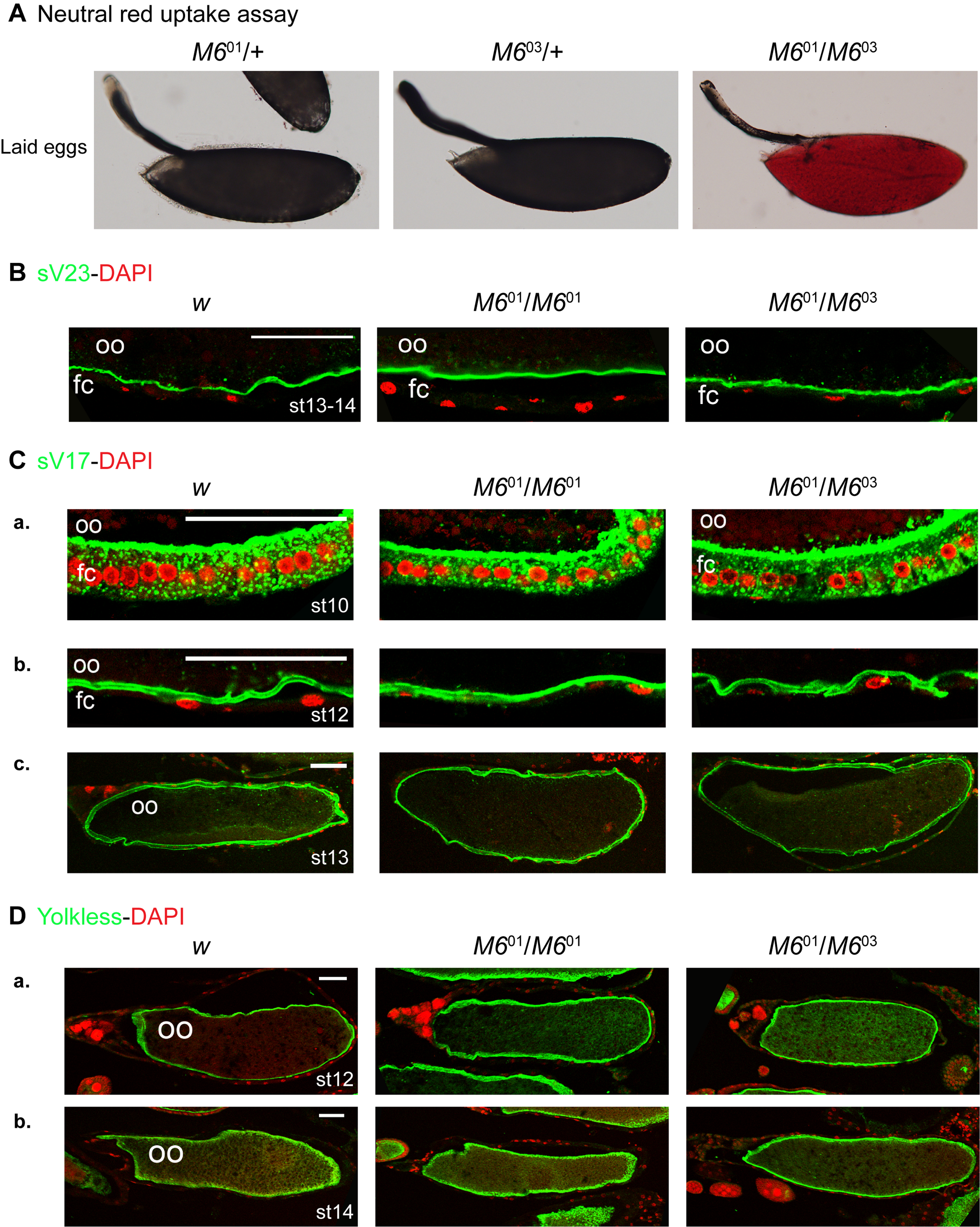

Supplement: Figure S3 — M6 01 and M6 01/ M6 03 egg chambers do not show altered localization of vitelline membrane proteins. (A) Eggs laid by heterozygote M6 01/+ and M6 03/+ and transheterozygote M6 01/M6 03 females were collected and incubated with the neutral red dye. All (100%) M6 01/M6 03 mutant eggs were permeable, which is taken as an indication of an abnormal vitelline membrane. (B–D) Immunofluorescence of wild type (w, left panel), M6 01 (middle panel) and M6 01/M6 03 (right panel) sectioned egg chambers with antibodies directed to sV23 (green, B), sV17 (green, C) and Yolkless (green, D). Numbers indicate egg chambers stages. Nuclei are visualized in red (DAPI). Projection of z-stacks are shown in (Cc.) and (Da,b). M6 mutant egg chambers displayed a continuous line of the vitelline membrane proteins (sV17 and sV23 staining), and no relocalization into the oocyte. Scale bar is 50 µm. Oo, oocyte; Fc, follicle. (TIF) [file pone.0019715.s003.tif]

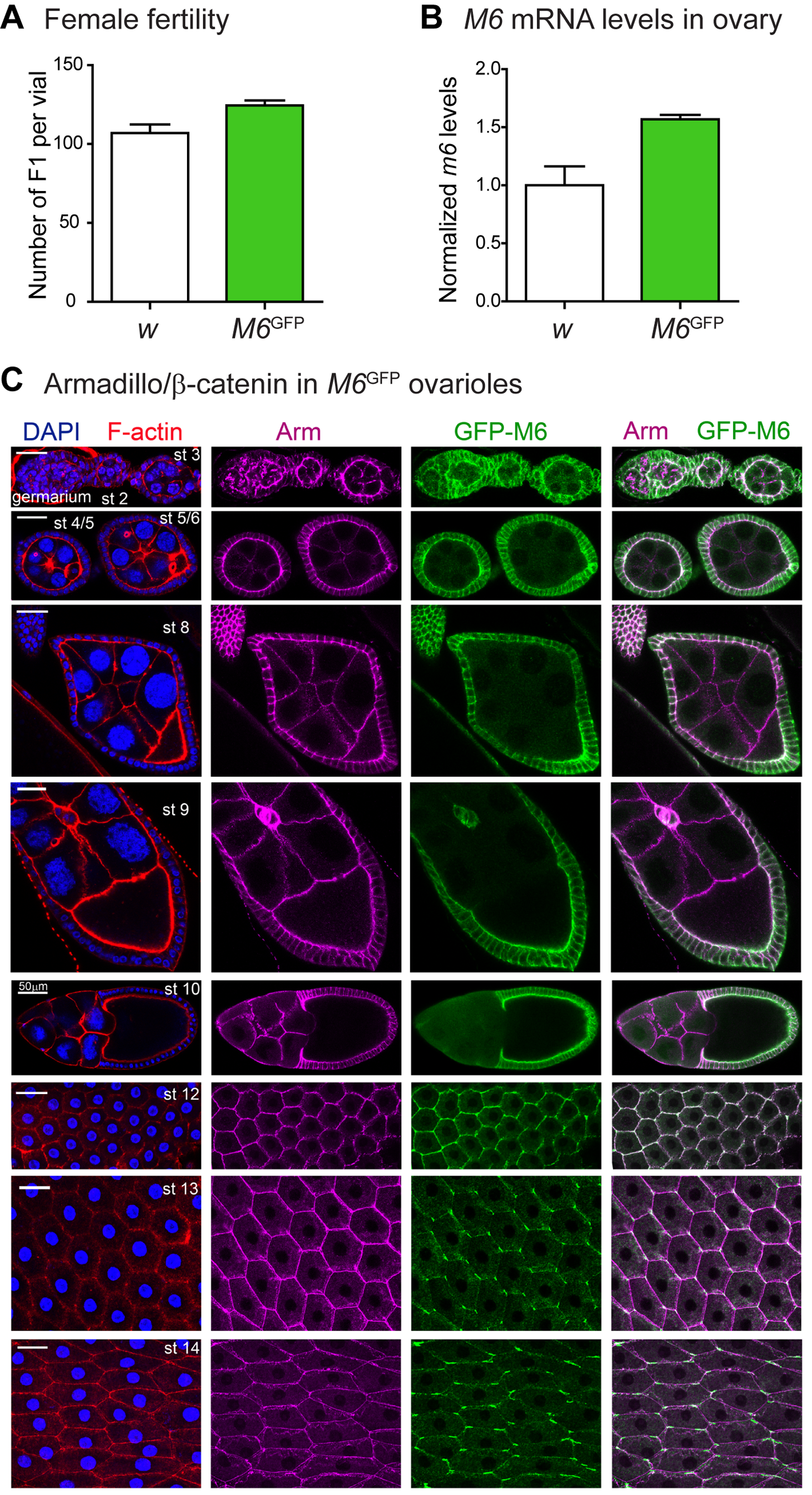

Supplement: Figure S4 — GFP-M6 colocalizes with Armadillo in the follicular epithelium. (A) Female fertility assessment of M6 GFP flies. Female fertility was measured as the number of offspring per vial obtained when crossed to wild type males. Mean ± s.e.m, n = 3; unpaired t-test with Welch correction, p<0.05. (B) M6 mRNA levels in ovaries was measured in wild type (w) and M6 GFP flies by RT-qPCR and normalized to Rp49 or gapdh. Statistical analysis included an unpaired t-test with Welch correction, p>0.05; mean ± s.e.m, n = 3. (C) Armadillo/β-catenin labels the membrane of follicle and nurse (to a lower extent) cells (magenta). GFP-M6 is localized to the membrane of the follicle cells throughout oogenesis in M6 GFP (green). Colocalization is shown on the right panel. Representative egg chambers are shown. F-actin was labeled with phalloin (red) and nuclei with DAPI (blue, left panels). Scale bar is 20 µm, unless otherwise indicated. (TIF) [file pone.0019715.s004.tif]

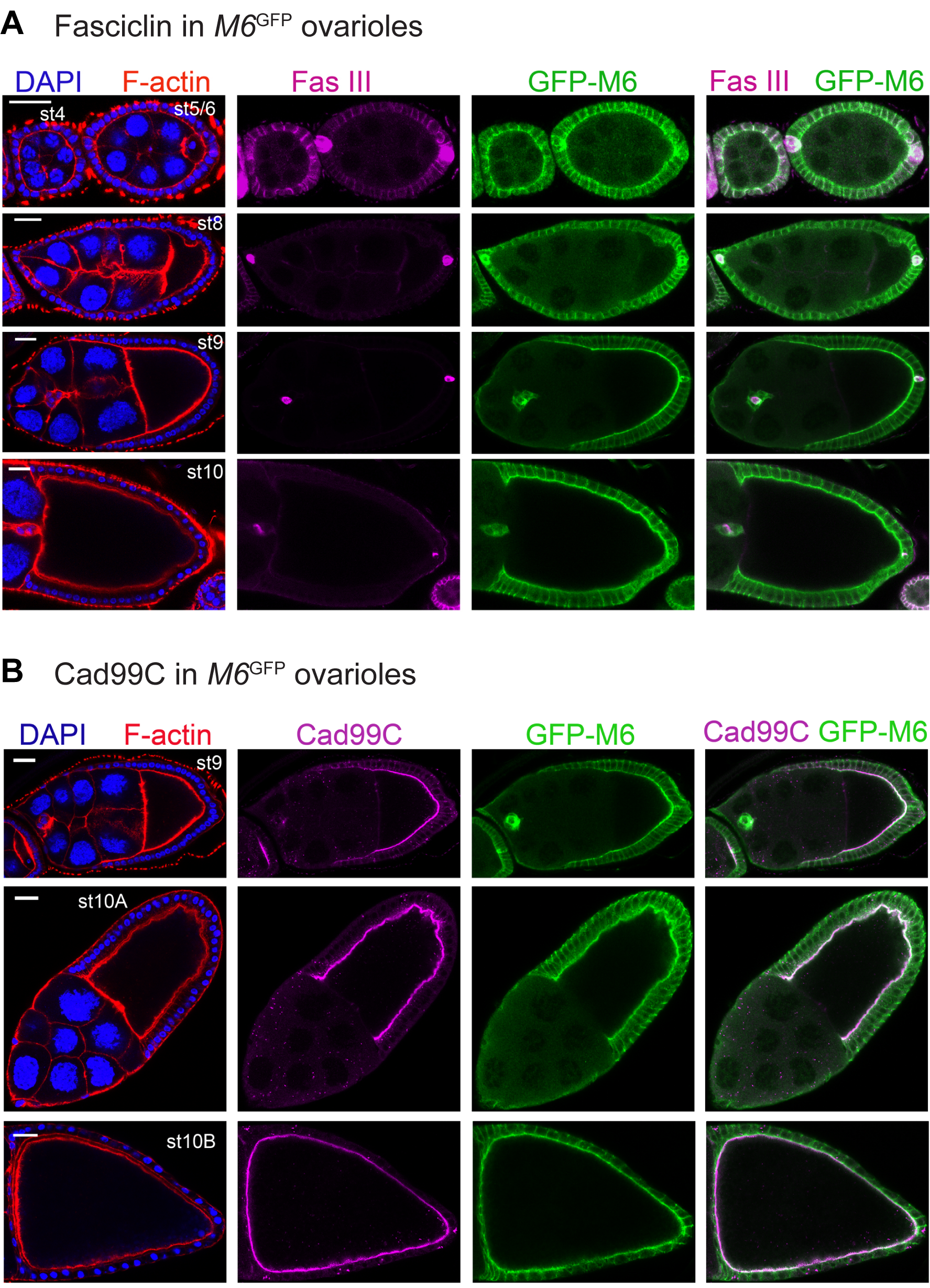

Supplement: Figure S5 — GFP-M6 colocalizes with Fasciclin and Cad99C. GFP-M6 (green) localizes to polar (also labeled with Fasciclin III, in magenta) and border cells (A) and to microvilli of the follicle cells labeled with anti-Cad99C antibodies (B, in magenta) in M6 GFP. Colocalization is shown on the right panel. Representative egg chambers are shown. F-actin was labeled with phalloidin (red) and nuclei with DAPI (blue, left panels). Scale bar is 20 µm. (TIF) [file pone.0019715.s005.tif]

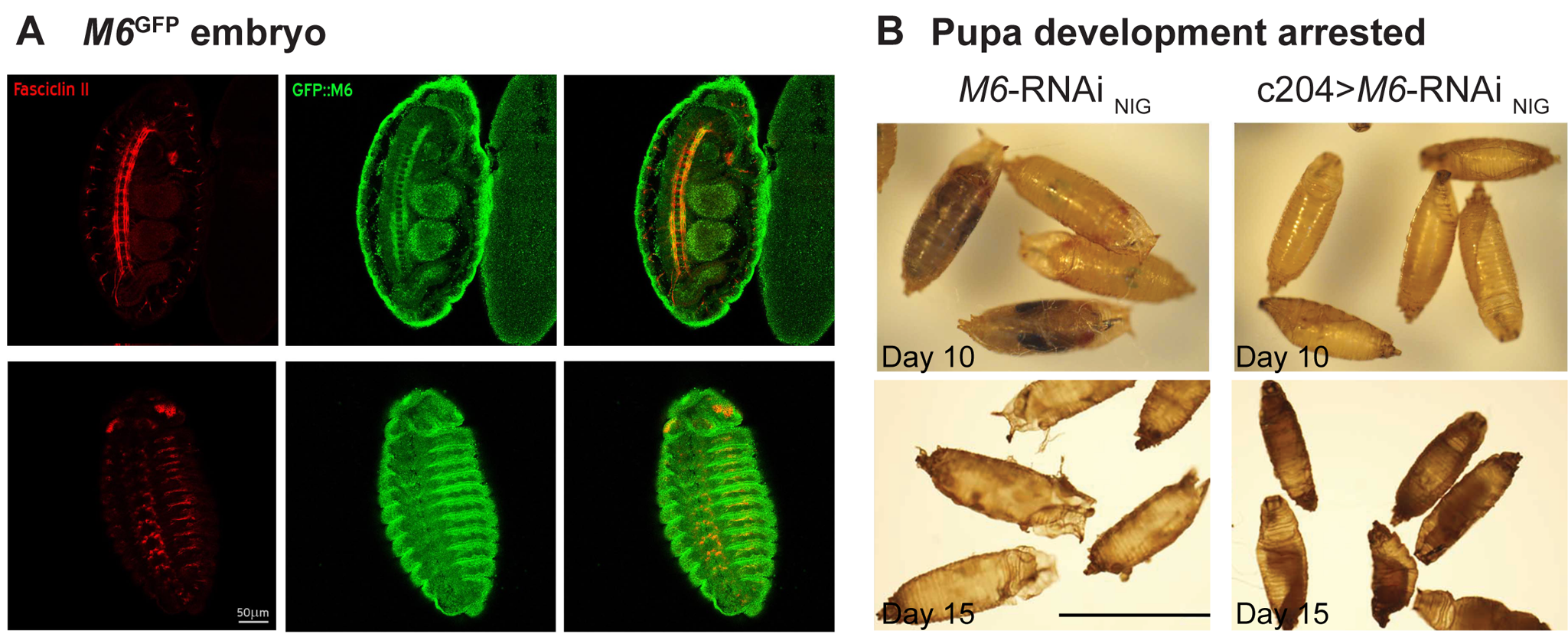

Supplement: Figure S6 — M6 requirement during development. (A) GFP-M6 is expressed in the embryo. Immunofluorescence images at different depths from the same M6 GFP embryo are presented in the upper and lower panels. Fasciclin II [red; anti-FasII MAb 1D4 (1∶5; DSHB)] labels longitudinal axon fascicles in the nervous system. GFP-M6 (green) is expressed in the longitudinal fascicles as shown by the colocalization (right panel), as well as in other tissues such as the epithelium (lower panel). Therefore, M6 knockdown in the embryo might be responsible for early lethality (see Table S2 for details). Scale bar is 50 µm. (B) When employing various enhancer traps reported to be expressed in the follicle cells to induce M6-RNAi, such as c204 and T155 (Bateman et al., 2001), an arrested pupae development was observed, suggesting that in addition to driving expression in follicle cells, those GAL4 drivers are also expressed at an unspecified tissue during larval/pupae formation. Thus, M6 deprivation during larval or pupae development stages abrogates metamorphosis (see also Table S2). Images of day 10 (upper panel) and 15 (lower panel) of Drosophila development at 25°C from flies bearing the enhancer traps c204 and the strong allele of M6-RNAi (C204>M6-RNAiNIG) and control flies (M6-RNAiNIG with no driver). Pupae arrest was detected during metamorphosis while control flies developed normally. Similar results were observed using T155. Scale bar is 2 mm. (TIF) [file pone.0019715.s006.tif]

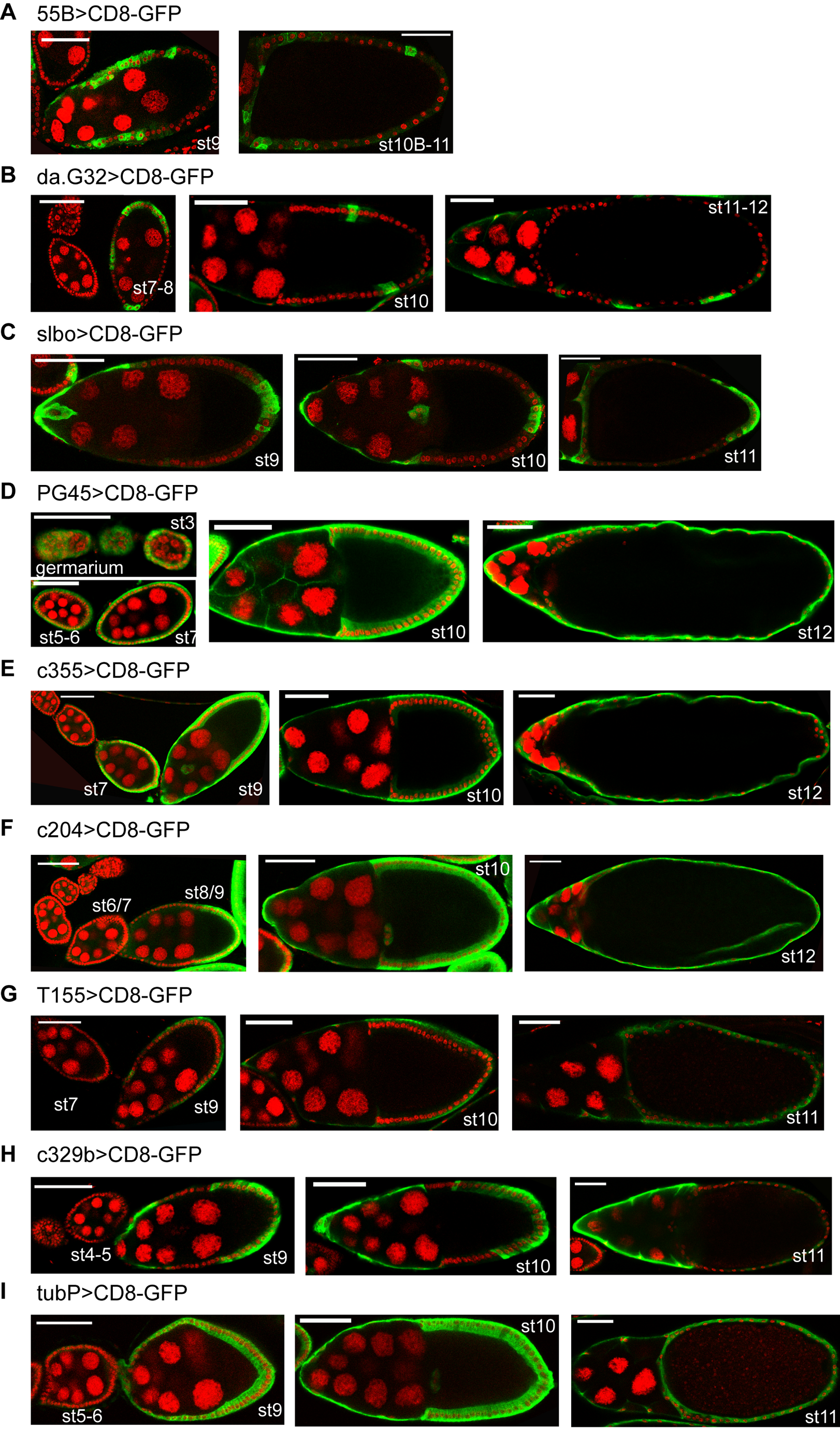

Supplement: Figure S7 — Follicular GAL4 drivers used to downregulate M6 in FC. Representative images of immunofluorescence of (A) 55B>CD8GFP, (B) da.G32>CD8GFP, (C) slbo>CD8GFP, (D) PG45>CD8GFP, (E) c355>CD8GFP, (F) c204>CD8GFP, (G) T155>CD8GFP, (H) c329b>CD8GFP and (I) tubP>CD8GFP ovarioles. GAL4 expression is revealed by CD8-GFP labels (green) in follicle cells. Nuclei were stained with propidium iodide or DAPI (red). PG45 expressed GAL4 in all follicle cells throughout oogenesis (D). Note GAL4 expression in subpopulation of follicle cells with different drivers (A,B,C and H). Interestingly, those follicular drivers (55B, da.Gal4, slbo.Gal4 and c329b) did not trigger any female sterility in the context of M6-RNAi, whereas PG45 did (see also Table S2). GAL4 is expressed in all follicle cells from st7 onwards (c355, (E)) and from st8/9 onwards (c204, (F)). The specific spatial or temporal expression profiles or GAL4 expression strength may account for the differences observed in female sterility phenotype when crossed to the M6-RNAi (see also Table S2). Representative egg chambers are shown. Scale bar is 50 µm. (TIF) [file pone.0019715.s007.tif]

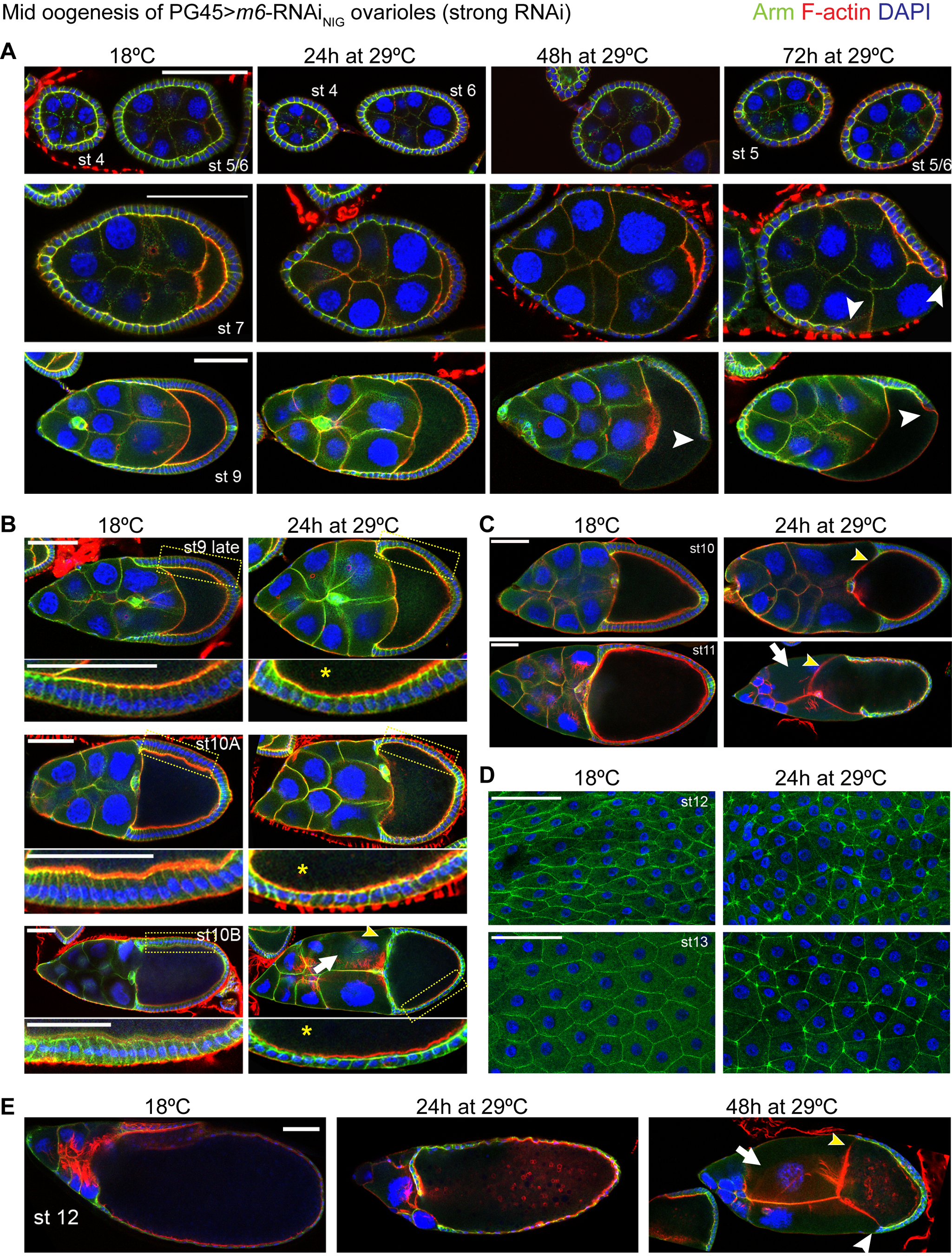

Supplement: Figure S8 — M6 downregulation in follicle cells induces a variety of defects in mid- and late oogenesis. Confocal images of representative egg chambers at different stages of oogenesis. Ovaries were dissected and stained with phalloidin (red), DAPI (blue) and Armadillo (green). PG45>M6-RNAiNIG (PG45; tub-GAL80ts; UAS-M6-RNAiNIG) flies were raised at 18°C and then adult females were transferred to 29°C for 24, 48 or 72 hours to induce M6 interference in the follicular epithelium or kept at 18°C as the non-induced control. Control females, M6-RNAiNIG (without PG45), shifted to 29°C for 72 hours were undistinguishable from non-induced controls (data not shown). Egg chambers corresponding to stages 4–7 and 9 (A), late stage 9, stage 10A and 10B (B), stages 10 and 11 (C), stages 12 and 13 (D) and stage 12 (E) are shown. (A) No morphological defects were observed at early stages of oogenesis. The presence of gaps in the follicular epithelium after 48 h of M6-RNAi induction became detectable from stage 8 onwards (indicated by white arrowheads), and from stage 7 onwards after 72 h of induction. Note defects in the FC shape. (B) The first defects observed after a 24 hour induction involved follicle cell morphology in egg chambers from mid-oogenesis, including a deficit in the thickness gradient of the posterior follicle cells in late stage 9 along with a failure in the columnar shape of the follicle cells in egg chambers reaching stage 10A. Defects in the overall shape of the follicle cells are indicated by yellow asterisks. Magnified views of the indicated areas are shown at the bottom of each egg chamber. White arrows highlight morphological hallmarks of the dumpless-like phenotype in st10B (i.e., disproportioned oocyte to nurse cell cellular size). (C) After a 24 hour induction, defects in centripetal migration were detected in stage 10–11 egg chambers as indicated by yellow arrowheads. This alteration might cause the dumpless-like phenotype(white arrow). (D) Disorganization of the f [file pone.0019715.s008.tif]
