# Supplementary material for: M6 Membrane Protein Plays an Essential Role in Drosophila Oogenesis
Source: PLoS One. 2011 May 16;6(5):e19715. doi: 10.1371/journal.pone.0019715 (PMC3095610; doi:10.1371/journal.pone.0019715)
Supplement: Table S1 — Primer sequences used in PCR. To characterize the M6 allelic series, PCR reactions on genomic DNA obtained from homozygous flies were carried out with primers directed to the 5′UTR of M6A flanking the original P element insertion. The E-GFP fusion to M6 was assessed by a Hemi-Nested PCR experiment employing cDNA from M6 GFP ovaries. Two rounds of PCR were performed in order to amplify isoforms with low representation. A second PCR experiment was carried out employing different pairs of primers. M6 mRNA isoforms tagged to the C-terminus of E-GFP were determined by sequence alignments (KAlign algorithm; www.ebi.ac.uk). The sequences of the expressed GFP::M6 proteins were predicted in silico. Primer sequences used in qPCR were designed using Primer Express 3.0 software (Applied Biosystems) and targeted to the 3′UTR of all M6 mRNA isoforms, glyceraldehyde phosphate dehydrogenase (gapdh) and ribosomal protein 49 (Rp49). To confirm M6 levels in the M6 allelic series a second pair of primers for M6 3′UTR was used. (DOC) [file pone.0019715.s009.doc]

Table S1: Primer sequences used in PCR.

| **Technique** | **Target** | **Primers** | **Primer sequences 5’-3’** |
| --- | --- | --- | --- |
| PCR | 5’UTR of *M6A* | Forward | TCGTTATTGTGTAGATACTATT |
|  |  | Reverse | CCGAAATGAAGTTATATCATTTAAGTGTTTC |
| Hemi-nested PCR | *gfp-M6* | Forward-1st round | AATTCCAACGCAACTAACAAATTG |
|  |  | Forward-2d round | TCATTTCGGAGACTGGTAACCAT |
|  |  | Reverse | CTGTACTCCAGCTCGTTCAGGTT |
| PCR | *gfp-M6* | Forward | ATGGTGAGCAAGGGCGAG |
|  |  | Reverse | TTGGGTTTCGTTGGAAGGTG |
| qPCR | *M6* | Forward-1 | ATCAATAAATCCATGCCAACACAAT |
|  |  | Reverse-1 | GCATTCGGCAATTCAGAAGAA |
| qPCR | *M6* | Forward-2 | GCACCATGTTTAAACTGCCAAAT |
|  |  | Reverse-2 | AAAAGTCAGTGGAACAGTACAGTAGATAAGA |
| qPCR | *Rp49* | Forward | GAGTTCTTGTAACGTGGTCGGAA |
|  |  | Reverse | AACTCGGCACTCGCACATC |
| qPCR | *gapdh* | Forward | TCCTGGGCTACACCGATGAG |
|  |  | Reverse | GCGTCGAACACAGACGAATG |
