# Supplementary material for: M6 Membrane Protein Plays an Essential Role in Drosophila Oogenesis
Source: PLoS One. 2011 May 16;6(5):e19715. doi: 10.1371/journal.pone.0019715 (PMC3095610; doi:10.1371/journal.pone.0019715)
Supplement: Table S2 — M6 knockdown triggers lethality during development and sterility in adult females. The table summarizes both the developmental arrest phenotype (middle column) and the female sterility phenotype (middle column) induced by M6-RNAi. Different GAL4 drivers and enhancer trap lines (left column) were used to express two independent UAS-M6-RNAi lines from the NIG-fly (strong allele) and VDRC (weak allele) stocks centers. Interference was amplified including UAS-Dicer2 (from VDRC). Early development arrest is triggered upon M6 downregulation. When pleitropic GAL4 drivers such as tubP-GAL4 [24], or follicular-specific GAL4 drivers such as the enhancer trap GAL4-daG32 [20] and PG45 [24], which are also expressed in the embryo, were used to trigger M6 interference, early lethality was observed. In every genetic combination (GAL4 driver>M6-RNAi) lethality became apparent at early stages during development, suggesting an essential role for M6 early on. This was also evidenced by the lethality of the M6 03 mutant and a number of M6 alleles generated by P element excision of the M6 01 mutant (data not shown). Some of the drivers required the addition of an allele of UAS-Dicer2 to trigger the same interference with both RNAi stocks (data not shown). Only complete female sterility was scored as female sterility. Although differences in the strength of the GAL4 drivers cannot be ruled out, M6 downregulation induced female sterility only when M6-RNAi was expressed in all follicle cells from st7 onwards (PG45, c355 and tubP-GAL4). Follicular GAL4 drivers were crossed to UAS-CD8GFP to check GAL4 expression in follicle cells (right column and Fig S7). Discrepancies from the literature were detected. (*) employing tub-GAL80TS to repress M6-RNAi through development at 18°C. Then, M6-RNAi was induced in young adult females at 29°C. (**) tested with M6-RNAiNIG. (***) tested with M6-RNAiVDRC FC for follicle cells. (DOC) [file pone.0019715.s010.doc]

**Table S2.** M6 knockdown triggers lethality during development and sterility in adult females.

| **GAL4 driver or enhancer trap** | **Lethality phenotype** | **Female sterility phenotype** | **GAL4 expression in egg chambers** |
| --- | --- | --- | --- |
|  | Driver crossed to UAS-*M6*-RNAi + UAS-*Dicer2* | | Driver crossed to UAS-CD8-GFP |
| 198-Y | No (**) | No | Undetectable in FC |
| e22C (en2.4GAL4) | No (**) | No | Undetectable in FC |
| 55B | No | No | Subpopulations of FC |
| da.G32 | Yes | No (*) | Subpopulations of FC |
| slbo | No | No | Subpopulations of FC |
| PG45 | Yes | Yes (*) | Strong in all FC, but lower in polar and border FC, until st13 |
| c355 | Yes (few escapers, ***) | Yes (*) | Strong in all FC, except polar and border FC, from st7 to st13 |
| c204 | Yes, pupae arrest (**) | No (*) | Strong in FC over the oocyte from st8 to st14 |
| T155 | Yes, pupae arrest (**) | No (***) | Mild in all FC from st9 to st11 |
| c329b | No (***) | No (***) | Strong in subpopulations of FC from st8 to st12 |
| tub-GAL4 | Yes | Yes (*) | Strong in all FC from st7 to st14 and mild in early stages |
